# Phenotypic deficits in the HIV-1 envelope are associated with the maturation of a V2-directed broadly neutralizing antibody lineage

**DOI:** 10.1371/journal.ppat.1006825

**Published:** 2018-01-25

**Authors:** Lucia Reh, Carsten Magnus, Claus Kadelka, Denise Kühnert, Therese Uhr, Jacqueline Weber, Lynn Morris, Penny L. Moore, Alexandra Trkola

**Affiliations:** 1 Institute of Medical Virology, University of Zürich, Zürich, Switzerland; 2 Department of Biosystems Science and Engineering, ETH Zurich, Basel, Switzerland; 3 Swiss Institute of Bioinformatics (SIB), Switzerland; 4 Division of Infectious Diseases, University Hospital Zürich, Zürich, Switzerland; 5 Centre for HIV and STIs, National Institute for Communicable Diseases (NICD), of the National Health Laboratory Service (NHLS), Johannesburg, South Africa; 6 Faculty of Health Sciences, University of the Witwatersrand, Johannesburg, South Africa; 7 Centre for the AIDS Programme of Research in South Africa (CAPRISA), University of KwaZulu-Natal, Durban, South Africa; Emory University, UNITED STATES

## Abstract

Broadly neutralizing antibodies (bnAbs) to HIV-1 can evolve after years of an iterative process of virus escape and antibody adaptation that HIV-1 vaccine design seeks to mimic. To enable this, properties that render HIV-1 envelopes (Env) capable of eliciting bnAb responses need to be defined. Here, we followed the evolution of the V2 apex directed bnAb lineage VRC26 in the HIV-1 subtype C superinfected donor CAP256 to investigate the phenotypic changes of the virus populations circulating before and during the early phases of bnAb induction. Longitudinal viruses that evolved from the VRC26-resistant primary infecting (PI) virus, the VRC26-sensitive superinfecting (SU) virus and ensuing PI-SU recombinants revealed substantial phenotypic changes in Env, with a switch in Env properties coinciding with early resistance to VRC26. Decreased sensitivity of SU-like viruses to VRC26 was linked with reduced infectivity, altered entry kinetics and lower sensitivity to neutralization after CD4 attachment. VRC26 maintained neutralization activity against cell-associated CAP256 virus, indicating that escape through the cell-cell transmission route is not a dominant escape pathway. Reduced fitness of the early escape variants and sustained sensitivity in cell-cell transmission are both features that limit virus replication, thereby impeding rapid escape. This supports a scenario where VRC26 allowed only partial viral escape for a prolonged period, possibly increasing the time window for bnAb maturation. Collectively, our data highlight the phenotypic plasticity of the HIV-1 Env in evading bnAb pressure and the need to consider phenotypic traits when selecting and designing Env immunogens. Combinations of Env variants with differential phenotypic patterns and bnAb sensitivity, as we describe here for CAP256, may maximize the potential for inducing bnAb responses by vaccination.

## Author summary

HIV-1 infected individuals rarely develop broadly neutralizing antibodies (bnAbs) that inhibit diverse HIV-1 subtypes. As activity against the majority of HIV-1 isolates is necessary for effective immunization against HIV-1, current vaccine development seeks to generate regimens that evoke bnAb responses. Delineating why bnAbs develop in certain cases of HIV-1 infection is thus of pivotal importance. In all infected individuals, HIV-1 inevitably escapes neutralizing antibody pressure and even the most potent bnAbs cannot clear the infection from the patient where they emerged. Yet, exactly this continuous interplay between virus escape and antibody maturation is believed to be crucial in the evolution of bnAbs, with virus escape variants containing modified HIV-1 envelope (Env) proteins, the target of neutralizing antibodies that in some cases can direct the immune response towards breadth. Identifying features of naturally occurring Env proteins that are involved in evoking bnAb responses is thus of high interest. Here, we analyzed Env features of virus isolates from donor CAP256 who developed the potent V2 apex bnAb VRC26, one of the current lead bnAbs for HIV-1 therapy and vaccine development. We show that the viral escape variants that appeared soon after the onset of the VRC26 response had highly altered Env protein properties that, in addition to reducing sensitivity to VRC26, affected their capacity to infect and altered entry dynamics. This highlights constrained viral escape pathways, but also features of VRC26 that may have prevented rapid escape. We postulate that this may have resulted in a prolonged circulation of partially VRC26 sensitive viruses, hence allowing the bnAb response to mature.

## Introduction

Broadly neutralizing antibodies (bnAbs) are a focus of HIV-1 vaccine development and passive immunization strategies [1–7]. Owing to the exceptionally potent and broad bnAbs that have been isolated over recent years [8–12], a wealth of information on their function has become available. However, factors that govern bnAb evolution in infection are not fully resolved, nor have current vaccine designs succeeded in eliciting bnAb responses. The expectations for vaccines are high as they will need to do substantially better than in infection where bnAbs only evolve in around 10–25% of HIV-1 infected individuals, with the most potent elite neutralizing antibodies restricted to approximately 1% of infections [13–16].

A number of parameters have been implicated in the development of neutralization breadth including the length of HIV-1 infection, high viral loads, virus diversity, CD4 T cell loss, involvement of regulatory T cell subsets, viral subtype and host factors including ethnicity and HLA genotype [13,16–30]. It has become clear that a tight interplay of antibody and virus escape variants directs antibody adaptation and diversification towards the generation of bnAbs [31–36]. Escape from even the most potent bnAbs appears inevitable, as all bnAbs identified to date have been isolated from individuals who ultimately failed to control viremia [13,18,37,38]. However, it is the continuous exposure to these gradually escaping viruses that appears to be key for the evolution of bnAbs.

Env variants that are capable of stimulating the germline ancestors of bnAbs as well as intermediate Env variants that steer antibody evolution to tolerate viral diversity, conferring breadth, are therefore urgently sought [23–25]. The phenotypic features of Env variants associated with the elicitation and maturation of breadth are largely unknown. Does the initial infecting strain harbor distinct features that enable it to more efficiently initiate bnAb precursors? [39,40] Are the same features relevant for the later evolving Env variants? Does incomplete escape, paired with replication in sites where antibody access is restricted, enable the virus to survive despite the presence of potent neutralization activity?

In the present study, we have addressed these questions by investigating the phenotypic plasticity of HIV-1 Env during bnAb development. As a model, we took the evolution of autologous viruses during the maturation of the potent CAP256-VRC26 bnAb lineage. The V2 specific CAP256-VRC26 bnAb lineage developed in a HIV-1 subtype C superinfected individual[33,41]. Frequent sampling and detailed analysis of this donor over several years has resulted in a large collection of both longitudinal Env variants and VRC26 bnAbs[33,41]. These co-evolved Env and VRC26 bnAbs therefore provide an ideal setting to explore phenotypic shifts in Env that may reveal insights into properties that are important for antibodies like VRC26 to develop. Comparing Env properties of the primary infecting (PI) virus, the superinfecting (SU) virus and their descendants circulating prior to and during the rise of the CAP256-VRC26 bnAb lineage, our study aimed to assess differential functional capacities that may have shaped the evolution of the CAP256-VRC26 bnAb lineage.

## Results

### Assessing the phenotypic plasticity of HIV-1 during CAP256-VRC26 bnAb induction

**Fig 1:**
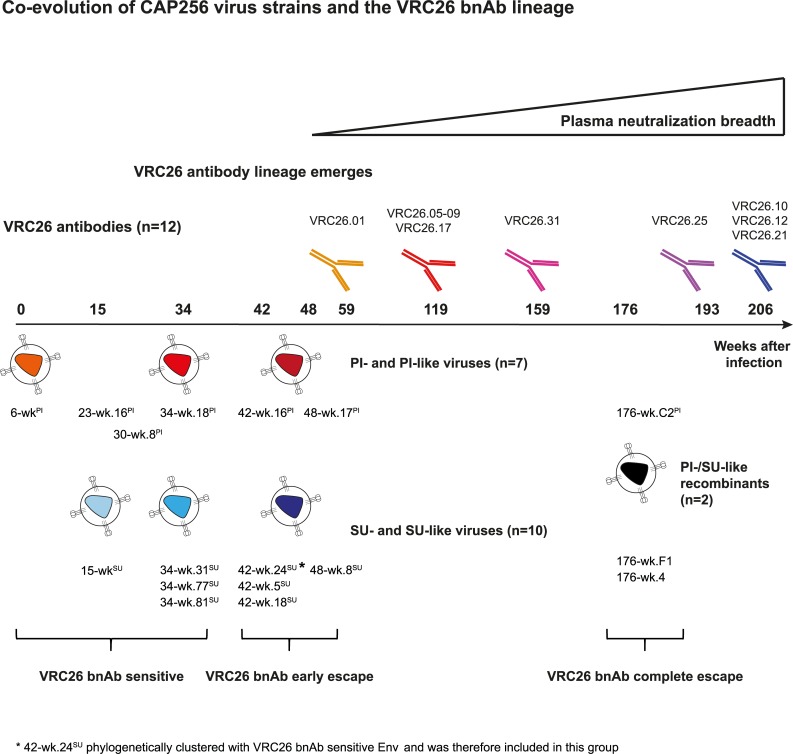
Co-evolution of CAP256 virus strains and the VRC26 bnAb lineage. Time line of the evolution of the CAP256 virus strains (PI-like, SU-like, PI/SU recombinants) and the VRC26 lineage bnAbs analyzed in the current study. Codes of bnAb and virus Env clones are indicated at the approximate time points of isolation. Classification of SU-like Env into *SU-like VRC26 sensitive*, *SU-like VRC26 early escape and SU-like VRC26 complete escape* is indicated.

The V2 apex targeting CAP256-VRC26 lineage emerged 30–34 weeks post infection (p.i.) after superinfection at week 15 (Fig 1 [34]). Plasma neutralization breadth developed around 48 weeks p.i. [42], and the first broad monoclonal antibodies were isolated from week 59 [33]. We included 17 longitudinal autologous viral envelopes (Env) from bnAb donor CAP256 (Fig 1, S1 Table) which circulated before and during the evolution of the bnAbs when the autologous viruses were still (partially) sensitive to the VRC26 bnAbs [33,34,41–43] and probed them against 12 members of the CAP256-VRC26 bnAb lineage with breadth varying from 7–63% (Fig 1, S2 Table). Fourteen of the CAP256 Env were isolated within the first 48 weeks of the infection and showed at least partial sensitivity to the VRC26 mAbs. These included the primary infecting (PI) virus (week 6) as well as the superinfecting (SU) virus (week 15) to which the bnAb response was mainly directed [33,34,42]. Later CAP256 viral variants were defined as PI-like, SU-like or PI/SU recombinant viruses according to their V1V2 sequence, which is the target of the VRC26 bnAbs [33,34] (S1 Table). In addition to the earlier variants up to week 48, we included three Env variants isolated at week 176 of infection, which had completely escaped the VRC26 lineage [33,42]. These Env were used to assess phenotypic functions that might impact antibody efficacy, namely viral infectivity, mode of transmission, sensitivity to neutralization, and entry kinetics. We specifically focused on defining properties for the SU virus variants, given the clear evidence that they induced the VRC26 lineage [33,41]. Due to the scope of the analyses, certain tests were restricted to the assessment of a smaller panel of Envs in which cases we prioritized the analyses of SU over PI viruses.

To assess the phenotypic properties of the CAP256 viruses, we first assessed their properties in free virus and cell-cell spread. HIV-1 has the ability to spread as free virus or through contacts between infected and uninfected target cells [44,45]. Most neutralizing antibodies (nAbs) including bnAbs, neutralize cell-cell transmission less effectively than free virus spread [46–53]. The loss of neutralizing activity (regardless of specificity) during cell-cell transmission is often substantial, decreasing bnAb activity 10–100 fold compared to free virus transmission [47,49–53]. Importantly, this reduced activity during cell-cell transmission can therefore substantially contribute to neutralization escape [47,52,54]. However, the VRC26 bnAbs [52], similar to MPER bnAbs [47,52], frequently retain comparable activity against heterologous HIV-1 strains in both transmission routes. Understanding how this capacity evolved in VRC26 is of interest as preserved activity in both modes of transmission will limit the emergence of escape variants [54] and would therefore be highly desirable for therapeutic and vaccine induced bnAbs.

**Fig 2:**
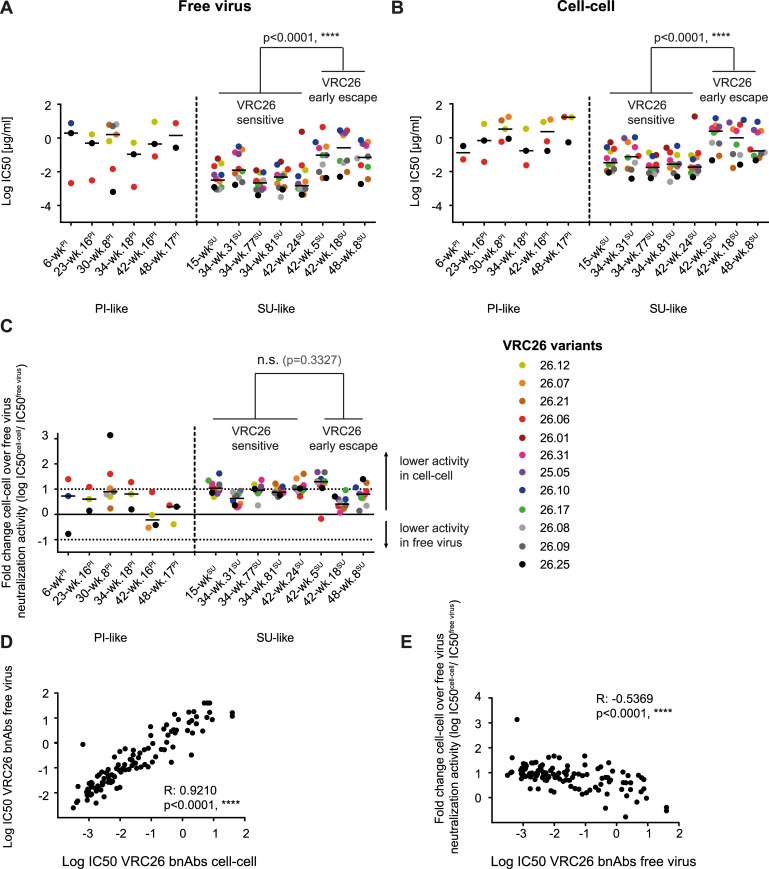
CAP256-VRC26 bnAbs maintain neutralization activity against cell-cell transmission of autologous viruses. **A** and **B:** 50% inhibitory concentrations (IC50 in μg/ml) of CAP256-VRC26 bnAbs against free virus (**A**) and cell-cell transmission (**B**) of indicated CAP256 virus strains are shown. Data are means of 2 to 4 independent experiments. Black lines depict the median IC50 of all sensitive antibody-virus combinations. *SU-like VRC26 sensitive* viruses were compared to *SU-like VRC26 early escape* viruses using the Mann-Whitney test. P values are indicated. **C:** The change in neutralization activity for cell-cell compared to free virus transmission is displayed as the log of ratio of IC50^cell-cell^/IC50^free-virus^ (log fold change). Zero denotes equal activity in both transmission pathways. 10-fold higher (log_10_ = 1) and lower (log_10_ = -1) IC50 in cell-cell over free virus transmission are indicated by dotted lines. Black lines show median fold change IC50^cell-cell^/IC50^free-virus^ for all sensitive VRC26/virus combinations. Different colors depict different bnAbs. **D+E:** Spearman correlation analyses of VRC26 bnAb neutralization in free virus and cell-cell transmission. R and p values are indicated. **D:** Neutralization activity in free virus and cell-cell spread (IC50 in μg/ml) of VRC26 bnAbs against autologous virus is tightly correlated (Spearman correlation, R: 0.9210, p<0.0001). **E:** Loss in cell-cell neutralization activity is associated with higher resistance of viruses to VRC26 bnAbs in the free virus pathway (Spearman correlation, R: -0.5369, p<0.0001).

To assess whether autologous cell-cell transmission was similarly sensitive to VRC26, and whether this was maintained over the course of infection, we assessed the selected 17 longitudinal CAP256 Env variants for neutralization sensitivity in the A3.01-CCR5 cell-based free virus and cell-cell transmission assays as described [52]. Week 176 viruses were fully resistant to VRC26 bnAbs (S2 Table) and were therefore not included in the comparison of free and cell-cell neutralization activity. As observed in free virus transmission (Fig 2A) [34,42,43], PI-like viruses were resistant to neutralization by most members of the VRC26 bnAb lineage during cell-cell transmission while SU-like viruses were sensitive (Fig 2B). In line with what we have reported previously for heterologous viruses [52], the VRC26 bnAbs showed a 10-fold mean activity loss across all VRC26 variants against the autologous virus strains during cell-cell transmission compared to free virus transmission (Fig 2B and 2C) with occasional virus-VRC26 bnAb combinations showing substantially lower and some higher activity losses in cell-cell than in free virus transmission (Fig 2C). Activity in cell-cell transmission was preserved across all VRC26 bnAb lineage members, regardless of the heterologous breadth of individual mAbs (S1A and S1B Fig) or the extent of their maturation, as measured by their level of somatic hypermutation (S1C and S1D Fig), suggesting that the preserved activity during cell-cell transmission may be a result of the specific mode of action of the VRC26 bnAbs.

Based on their sensitivity to VRC26 bnAbs, SU-like viruses were grouped into *SU-like VRC26 sensitive* and *SU-like VRC26 early escape* viruses. Later SU-like viruses (week 42–48) were significantly less sensitive to VRC26 inhibition than early SU-like viruses (week 15–34) in both transmission pathways except for clone 42-wk.24^SU^ which phylogenetically clustered with sensitive Env isolated at week 34 post infection (S2 Fig). Across the longitudinal PI and SU-like viruses, we observed similar antibody activity during free virus and cell-cell transmission (Fig 2A–2D) suggesting that cell-cell transmission may not have been a preferential escape pathway of the autologous virus from VRC26 pressure.

**Fig 3:**
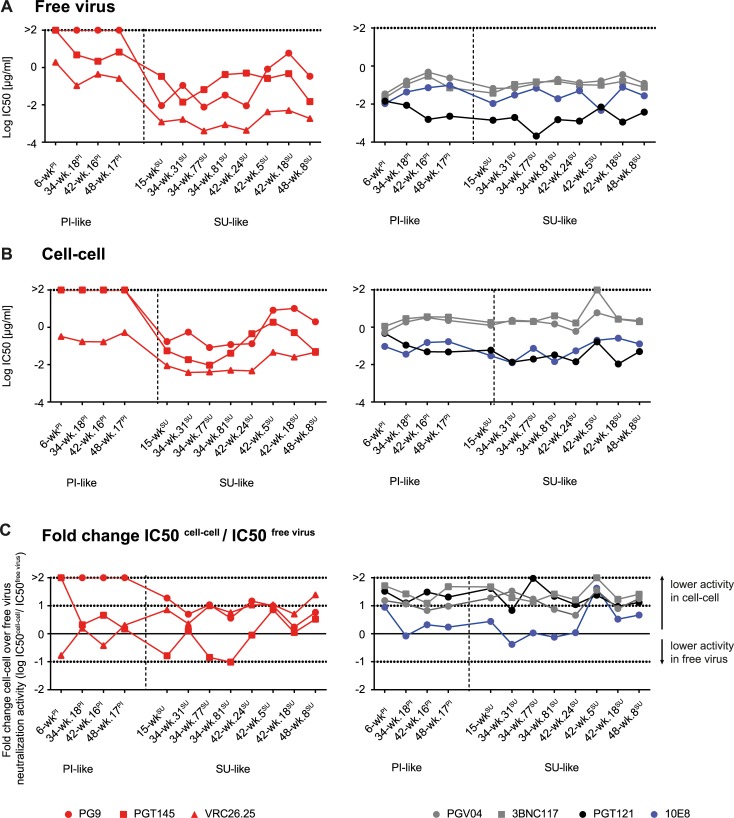
**Heterologous bnAbs exhibit different neutralization efficiencies in CAP256 Env free virus and cell-cell transmission A** and **B:** IC50 (in μg/ml) of heterologous bnAbs targeting the V2 apex (red), the CD4 binding site (grey), the V3 glycan supersite (black) or the MPER region (blue) against free virus (**A**) and cell-cell transmission (**B**) are shown. Data are means of 2 to 4 independent experiments. **C**: The change in neutralization activity for cell-cell compared to free virus transmission is displayed as the log of the ratio of IC50^cell-cell^/IC50^free-virus^ (log fold change). Zero denotes equal activity in both transmission pathways. 10-fold higher (log_10_ = 1) and lower (log_10_ = -1) IC50 in cell-cell over free virus transmission are indicated by dotted lines.

We therefore investigated if the high activity of the VRC26 lineage against cell-cell spread is unique to VRC26 or a feature common to V2 apex-directed bnAbs. We compared inhibition of free virus and cell-cell transmission of the CAP256 virus panel by bnAbs targeting the CD4 binding site (CD4bs; PGV04 [27] and 3BNC117 [55]), the V3 glycan supersite (PGT121 [56]), the V2 apex (PG9 [57] and PGT145 [56]) and the MPER region (10E8 [58]) with VRC26.25, the broadest and most potent VRC26 variant (Fig 3).

Increased resistance of CAP256 viruses to VRC26 was mirrored by a significantly higher resistance of both PI and SU-like viruses to free virus neutralization by PG9 and PGT145 (Mann-Whitney Test, p<0.0001), highlighting the similar modes of action of these V2 apex bnAbs (Fig 3A and 3B; [42]). While SU-like viruses up to week 48 were neutralized by the V2 glycan bnAbs, PI-like Env were highly resistant in free virus infection but even more so in cell-cell transmission where both PG9 and PGT145 completely lacked activity (Fig 3A and 3B). Sensitivity to the CD4bs, V3 glycan and MPER bnAbs did not fluctuate substantially in CAP256 viruses over time in either pathway (S3 Table). While the difference of SU-like virus 42-wk.5^SU^ cell-cell neutralization sensitivity to the other strains did not reach statistically significance, 42-wk.5^SU^ had notably the highest loss in cell-cell neutralization sensitivity for all bnAbs including the V2 glycan bnAbs. This may suggests that 42-wk.5^SU^ either adopted a specific Env conformation that occludes mAb access during cell-cell transmission for a wide spectrum of mAb specificities, or that this virus is particularly well adapted for cell-cell spread. In line with previous observations [47,52], MPER bnAb 10E8 displayed preserved neutralization activity against most CAP256 viruses during cell-cell transmission similar to the V2 glycan bnAbs (Fig 3). CD4bs and V3-directed bnAbs, however, showed a significantly lower activity during cell-cell transmission compared to V2- and MPER-directed bnAbs (Fig 3; Mann-Whitney Test of fold change IC50s of CD4bs- and V3 bnAbs versus V2- and MPER bnAbs, p<0.0001). Collectively, this suggests that the capacity to retain activity in cell-cell transmission is linked to the bnAb’s specificity and its mode of action.

### Early VRC26 escape results in virus variants with reduced entry fitness

**Fig 4:**
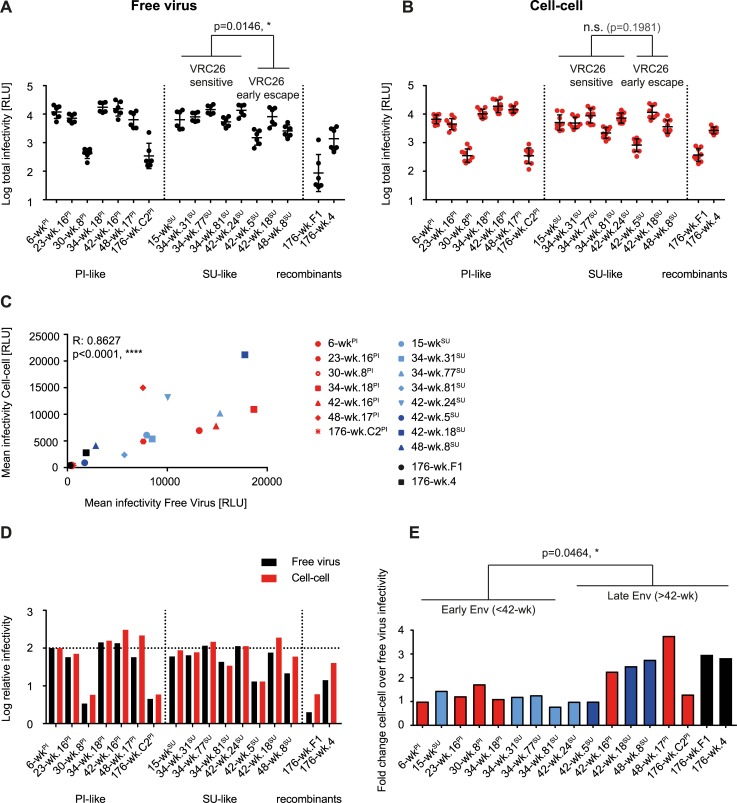
Early VRC26 escape results in virus variants with reduced entry fitness. **A** and **B:** Replicative capacity for CAP256 Env reporter pseudoviruses was tested by infecting A3.01-CCR5 cells either with free virus preparations (**A**) or transfected 293-T cells to probe cell-cell transmission (**B**). Infectivity for free virus (**A**, black) and cell-cell transmission (**B**, red) is displayed in relative light units (RLU). Means of two (free virus) to three (cell-cell) independent experiments in duplicates with SD are shown. *SU-like VRC26 sensitive* viruses were compared to *SU-like VRC26 early escape* viruses using the Mann-Whitney test. P values are indicated. **C:** Correlation between free virus and cell-cell infectivity were determined by Spearman correlation on untransformed data sets. R and p values are indicated. PI-like, *SU-like VRC26 sensitive*, *SU-like VRC26 early escape* and PI/SU recombinant viruses are marked in red, light blue, dark blue and black respectively. **D:** Results of the free virus (black) and cell-cell (red) infectivity assay were normalized to the PI-virus, 6-wk^PI^, and are indicated as relative infectivity. Relative means are shown. **E:** The ratios of relative infectivity values for cell-cell and free virus transmission are depicted. Early viruses (wk 6 to wk 34) were compared to late viruses (wk 42 to wk 176) using the Mann-Whitney test. The P value is indicated. PI-like, *SU-like VRC26 sensitive*, *SU-like VRC26 early escape* and PI/SU recombinant viruses are marked in red, light blue, dark blue and black respectively.

We compared Env functionality of the longitudinal CAP256 virus panel in free virus (Fig 4A) and cell-cell transmission (Fig 4B), and detected substantial variability in Env infectivity of PI and SU-like viruses in both transmission modes. High variability was even observed for Env variants from the same time point, as exemplified by Env clones from week 42 (Fig 4A and 4B). Overall, early escape viruses exhibited a significantly reduced entry fitness compared to VRC26 sensitive SU-like viruses in free virus but not cell-cell transmission. Interestingly, in both modes of transmission, the 176 week viruses showed significantly reduced (Mann-Whitney test, p<0.0001) entry capacity compared to earlier variants suggesting an eventual fitness cost for escape from VRC26 bnAbs. Although we have no formal proof at this stage for a direct causality, it is intriguing to note that the CAP256 donor experienced reduced viral loads at later time points that could be the result of decreased viral fitness [42].

The free virus and cell-cell infection assay systems require distinct culture conditions and therefore do not allow for a direct quantitative comparison of virus infectivity but provide a means for relative measures and comparison of patterns across pathways. Analyzing the relative infectivity of longitudinal CAP256 Env, we found that infectivity in free virus and cell-cell transmission was tightly linked, indicating that enhanced cell-cell transmission did not compensate for substantial defects in replication observed in free virus transmission (Fig 4A, 4B and 4C).

To obtain a more direct comparison of the infectivity patterns, we normalized all results to the PI virus (Fig 4D). With one exception (34-wk.81^SU^), the relative infectivity of viruses in the cell-cell format was comparable (+/- 10%) or higher than in free virus transmission across all three virus groups (PI-like, SU-like, PI/SU recombinants; Fig 4D). Interestingly, clone 42-wk.5^SU^, a clone from the onset of VRC26 escape (Fig 1), which displayed higher resistance to neutralization in cell-cell transmission (Fig 2), showed a significantly lower entry fitness than the other Env variants in both transmission pathways (Mann-Whitney test, p<0.0001). Thus, neutralization escape from VRC26 bnAbs coincided with loss in neutralization sensitivity across different bnAb specificities in cell-cell transmission for this Env variant but at the same time at a reduced entry capacity. Overall, cell-cell transmission efficacy was significantly higher amongst later evolving PI and SU variants (Fig 4E) and often coincided with low infectivity (Fig 4A and 4B). This may indicate that cell-cell transmission, while not fully compensating for entry defects, allowed for better replication for the infectivity impaired escape variants that emerged in response to VRC26 and the non-VRC26 autologous nAbs.

**Fig 5:**
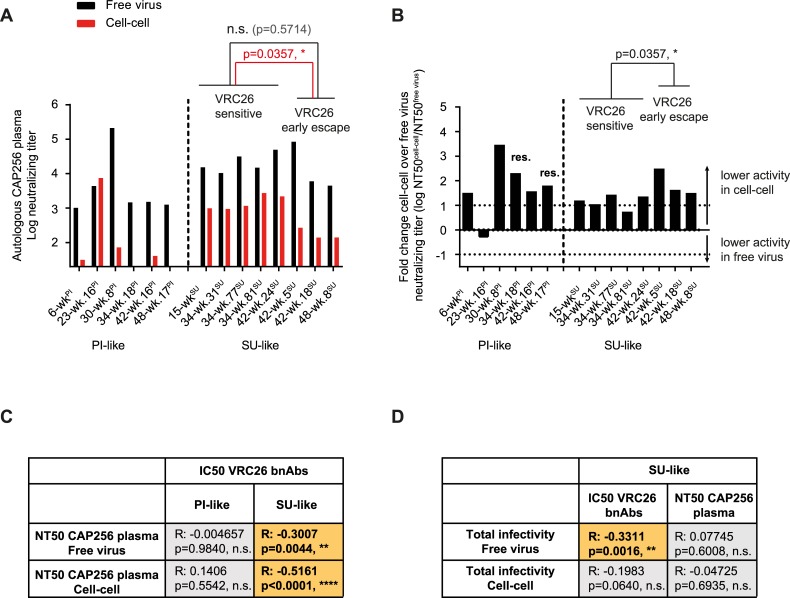
Activity of autologous plasma against cell-cell transmission of CAP256 viruses is strongly driven by VRC26 bnAb activity. **A:** 50% neutralizing titers (in plasma dilution) of autologous CAP256 plasma from week 145 p.i. against free virus (black) and cell-cell transmission (red) of CAP256 virus strains is depicted. Data are means of 2 to 3 independent experiments. Inhibition of *SU-like VRC26 sensitive viruses* and *SU-like VRC26 early escape* viruses in free virus and cell-cell transmission were compared using the Mann-Whitney test. P values are indicated in black and red respectively. **B:** The change in neutralization activity for cell-cell compared to free virus transmission is shown as the ratio of NT50^free-virus^/NT50^cell-cell^ (Fold change Neutralizing titer). Zero denotes equal activity in both transmission pathways. A 10-fold higher activity in free virus over cell-cell transmission reflects a log_10_ = 1, a 10-fold lower activity a log_10_ = -1 as indicated by dotted lines. Viruses that were not neutralized to 50% at the half lowest plasma concentration were denoted as resistant (res.) and their fold changes were estimated. **C:** Interrelations of neutralizing titers for plasma and IC50s for bnAb neutralization were determined separately for PI-like and SU-like viruses during free virus and cell-cell transmission. **D:** Interrelations of virus infectivity in free virus and cell-cell transmission, IC50s and neutralizing titers (NT50) were determined for SU-like viruses. **C+D:** Spearman correlations on untransformed data sets were used, R and p values are indicated. Orange fields negative correlations. N.s. denotes no significance.

To probe the impact of the polyclonal autologous neutralization response on virus evolution, we determined the sensitivity of CAP256 viruses in both transmission pathways to autologous plasma collected at week 145 p.i. (Fig 5A) which had been previously shown to have the maximum neutralization titer against heterologous viruses [33]. Viruses from week 176 were not included in this analysis, as they are resistant to week 145 plasma [42]. While both PI and SU-like viruses were sensitive to the autologous plasma in free virus transmission, titers were significantly higher for the SU-like viruses which were also significantly better neutralized during cell-cell transmission (Mann-Whitney test, p = 0.0426 for free virus and cell-cell), highlighting the dominance of VRC26 bnAbs in the autologous plasma. As observed for VRC26 bnAb neutralization, *SU-like VRC26 early escape* viruses were less sensitive to week 145 plasma inhibition than *early SU-like viruses*. However, differences were only significant for the cell-cell but not the free virus transmission pathway. (Fig 5A). In contrast, all but one PI-like virus showed high resistance to plasma antibodies during cell-cell transmission (Fig 5A and 5B). Furthermore, neutralization activity of VRC26 bnAbs and plasma against SU but not PI viruses correlated during free virus and cell-cell transmission (Fig 5A and 5C and S3A Fig), confirming that neutralization of PI viruses by the autologous plasma is mediated by other nAb specificities [42] that are presumably not active in neutralizing cell-cell transmission. A central question in understanding escape from neutralization is the consequence of escape mutations on Env fitness. Interestingly, increased VRC26 resistance was associated with free virus but not cell-cell infectivity loss (Fig 5D, S3B Fig). This is in line with the observation that late SU-like viruses maintain higher cell-cell than free-virus infectivity (Fig 4) paired with the slightly reduced cell-cell neutralization by VRC26 (Fig 2). Importantly, our findings highlight that in the early phase of VRC26 bnAb escape that we investigated here, virus variants with decreased fitness can emerge.

### CAP256 virus evolution results in altered entry kinetics

**Fig 6:**
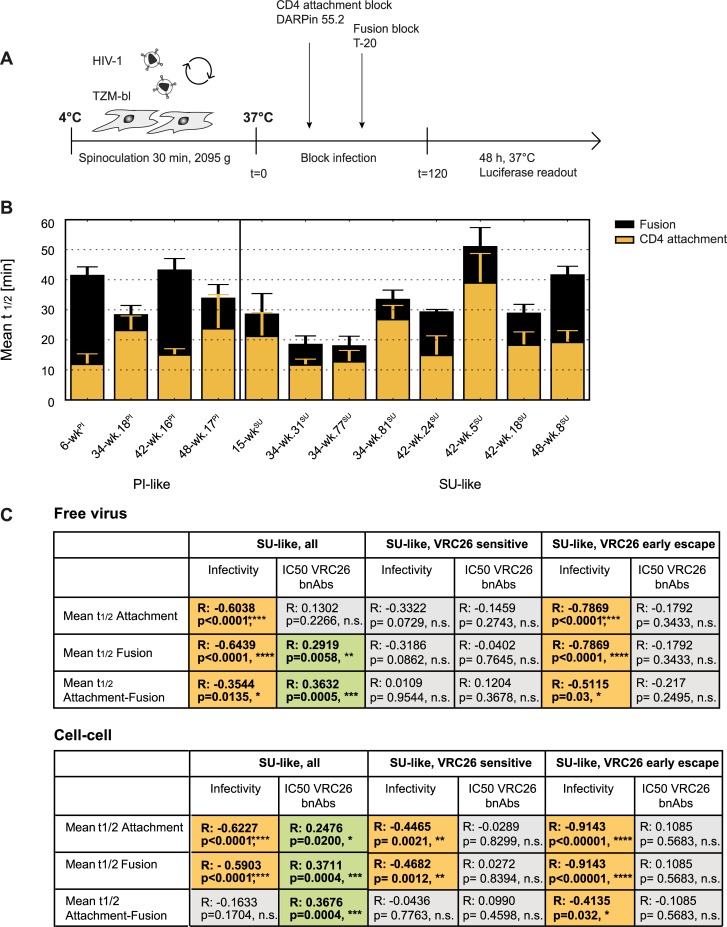
Virus evolution alters the entry kinetics of autologous CAP256 viruses. **A:** Schematic presentation of the free virus entry kinetic assay on TZM-bl target cells testing the half-maximal time to CD4-attachment and fusion. **B:** Mean half-maximal times of infection (t_1/2_) to CD4 attachment (orange) and virus fusion (black) of 2 to 4 independent experiments are displayed. **C:** Interrelations of IC50s (in μg/ml) for VRC26 bnAb neutralization, viral infectivity and mean half-maximal time (t_1/2_) to CD4-attachment, fusion and from CD4 attachment to fusion were determined for viruses analyzed in Fig 6B and displayed for all SU-like, *SU-like VRC26 sensitive* and *SU-like VRC26 early escape* viruses for both free virus and cell-cell transmission. Spearman correlation on untransformed data sets are shown with R and p values. Green fields denote positive, orange fields negative correlations. n.s. indicates no significance.

Our analyses thus far highlighted the importance of entry fitness differences during virus escape. To obtain mechanistic insights into which aspects of the entry process are linked with gains and losses in infectivity, we examined the entry kinetics of selected PI-like and SU-like viruses in an inhibitor time-of addition experiment as previously described [59,60] (Fig 6A). As this assay allows only for the analysis of a restricted number of Env at the same time, we focused on the SU-like Env to better study the phenotypic evolution of these autologous viruses. We analyzed the kinetics of CD4 attachment (blocked by the CD4 agent DARPin 55.2 [61]) and fusion (blocked by the fusion inhibitor T-20 [62]). Infection time courses for both entry steps were established by adding the respective inhibitors at distinct time points from 5 to 120 min. The mean half-maximal time (mean t_1/2_) required for viruses to progress with the entry process beyond these two steps were calculated from fitted curves (Fig 6B, S4 Fig) and time intervals between three different stages of the entry process (CD4 attachment, progress from CD4 attachment to fusion, and fusion) were compared (S5 Fig).

Amongst PI-like viruses, the PI virus (6-wk^PI^) and PI-like clone 42-wk.16^PI^ engaged CD4 rapidly but required an extended time period to progress to fusion and establish infection. PI-like viruses 34-wk.18^PI^ and 48-wk.17^PI^ showed a different entry phenotype with a trend to less rapid CD4 engagement and faster progression towards fusion narrowing the time window between completion of CD4 binding and fusion (Fig 6B) which, however, did not reach significance. A similar prolonged progression from CD4 binding to fusion was also seen for later SU-like viruses from week 42 onwards, which was significantly slower compared to earlier SU virus variants (Mann-Whitney test, p = 0.0286).

We hypothesized that these differences in entry kinetics could be linked with VRC26 escape and changes in infectivity. To probe this, we compared associations of entry kinetic parameters with neutralization sensitivity to VRC26 and infectivity, both in free virus and cell-cell transmission (Fig 6C, S6 Fig). In line with the high resistance of PI-like viruses to the VRC26 bnAb lineage, overall no relevant association between entry kinetics and sensitivity to the VRC26 bnAbs was observed (S6 Fig). SU-like viruses, however, showed a very interesting evolution in their kinetic properties. Prolonged entry kinetics for attachment and fusion were highly significantly linked with lower infectivity, both in free virus and cell-cell transmission. This pattern was largely driven by the *SU-like VRC26 early escape* variants. Indeed, prolonged entry kinetics correlated with increased VRC26 resistance in both, free virus and cell-cell transmission, confirming that the acquisition of mutations conferring increasing resistance to the VRC26 bnAb lineage were associated with an altered entry phenotype and fitness loss (Fig 6C, S6 Fig).

### CAP256-VRC26 bnAbs can act both pre- and post-CD4 attachment with high activity

**Fig 7:**
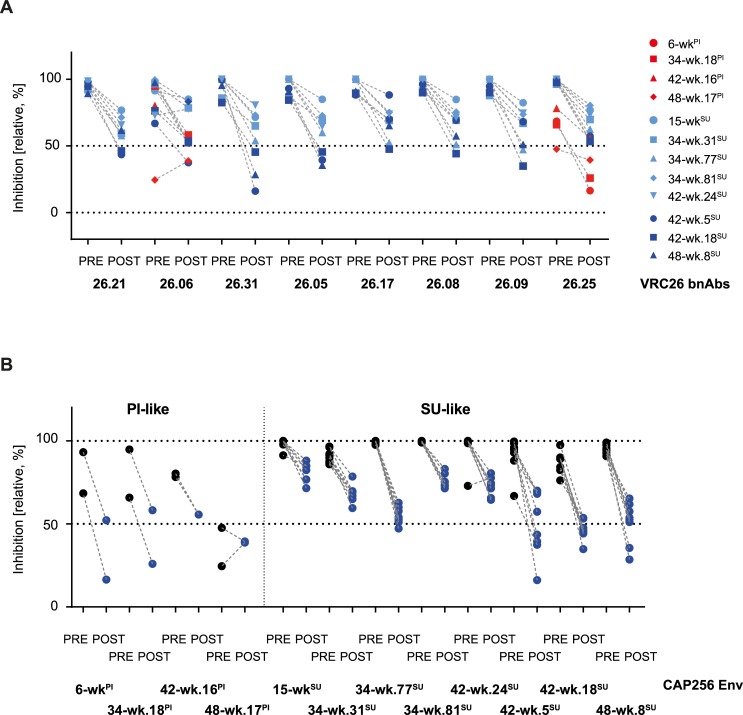
CAP256-VRC26 bnAbs display high activities at the pre- and post-CD4 attachment step. **A** and **B:** Pre- and post- CD4 attachment activities of the VRC26 bnAbs are displayed relative to the total activity of each bnAb-virus combination. Data were derived from two independent experiments **A:** Data displayed are sorted by bnAbs, PI-like, *SU-like VRC26 sensitive* and *SU-like VRC26 early escape* viruses are marked in red, light blue and dark blue respectively. **B:** Same data as in A, sorted by virus strains. Pre- and post-attachment activity are shown in black or blue respectively.

Interestingly, the time to CD4 engagement had no impact on free virus VRC26 potency and was also the weakest influence for cell-cell inhibition (Fig 6C), suggesting that VRC26 may not depend on a rapid binding prior to CD4 attachment in order to neutralize. We thus directly explored the efficacy of VRC26 against free virus before and after CD4 engagement for a selection of Env variants, focusing again on the SU-like autologous viruses (Fig 7). To distinguish pre and post CD4 activity, we synchronized infection by spinoculation at 23°C, a temperature that allows for virus binding to CD4 but not for fusion, and added the bnAbs either before or after CD4 attachment [47,52].

**Fig 8:**
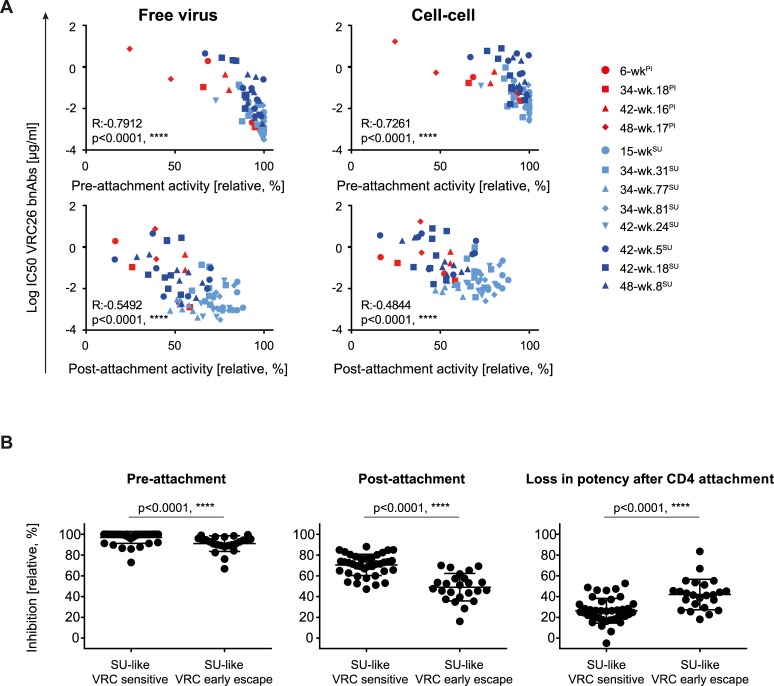
*SU-like sensitive* and *SU-like VRC26 early escape* viruses are differently neutralized post CD4 attachment. **A**: Interdependencies of pre- or post-attachment activity and IC50 for free virus (left) or cell-cell neutralization (right). Spearman correlations on untransformed data sets with R and p values are shown. Neutralization data are derived from data sets depicted in Fig 2, pre- and post- attachment data are derived from data sets depicted in Fig 7. PI-like, *SU-like VRC26 sensitive* and *SU-like VRC26 early escape* viruses are marked in red, light blue and dark blue respectively. **B:** Pre- and post-attachment inhibition and loss in activity after CD4 attachment based on data depicted in Fig 7 were compared for *SU-like VRC26 sensitive* and *SU-like early escape* viruses using the Mann-Whitney test. P values are indicated.

Individual virus-VRC26 bnAb combinations differed in pre- and post-attachment activity (Fig 7A and 7B) but overall, sensitivity in free virus and cell-cell transmission correlated with pre- and post-attachment activity for PI-like and SU-like viruses (Fig 8A). Most intriguingly, the *SU-like VRC26 sensitive* viruses were significantly better neutralized during both pre- and post-attachment (Figs 7B and 8B; Mann-Whitney test, p<0.0001). This ability to access their epitope both on the native trimer and post-CD4 triggering allows for a prolonged window of action that may thus contribute to the bnAbs’ potency and breadth for both viral transmission routes. For *SU-like VRC26 early escape* viruses, however, post-attachment neutralization decreased to a significantly greater extent (Mann-Whitney test, p<0.0001) compared to *SU-like VRC26 sensitive* viruses (Figs 7B and 8B), suggesting that resistance conferring mutations may have a more pronounced effect on the CD4 bound conformation of the CAP256 Env.

Considering all phenotypic traits we investigated, our analyses lead us to suggest that an initial pressure on free virus might have resulted in the generation of escape variants with altered entry properties. This first wave of partial escape from VRC26 might have led to a reduced entry capacity that was linked with altered entry kinetics and a more pronounced decrease in post-attachment activity of the VRC26 bnAb lineage.

## Discussion

A continuous, tight interplay between the HIV-1 envelope (Env) antibody response and virus escape is a fundamental component of bnAb development [31,32,34,35,41]. Consequently, elucidation of escape pathways can provide valuable insights for bnAb based vaccine design. Viral escape leads to the formation of new Env variants that affinity mature the bnAb response. Deciphering which Env variants were instrumental in shaping the bnAb response in natural infection will thus help to inform immunogen design. To date, we lack criteria that allow for pinpointing these Env variants amongst the wide spectrum of quasispecies that evolve during virus escape. Knowing which phenotypic features were preserved or alternatively lost during escape evolution will allow to select Env variants not only based on epitope variation, but also on phenotypic traits that may be relevant for epitope exposure, virus survival and antibody efficacy. In the present study, we explore phenotypic traits of the virus Env during bnAb evolution to distinguish Env properties that may have influenced bnAb development or resulted from its action. We do this by studying longitudinal Env and bnAb variants available from donor CAP256 who developed the potent V2 apex VRC26 bnAb lineage [33,34,41,42].

Although a range of host and viral factors that contribute to bnAb development have been identified [13,16–30], we currently do not know to what extent a given Env will steer the antibody response towards bnAb development. That *env* genes are not equally effective in inducing bnAbs is widely assumed and we have recently shown this in a survey of neutralization breadth in close to 4500 individuals where we observed higher frequencies of CD4bs bnAbs amongst HIV-1 subtype B infections while V2 apex bnAbs proved more frequent in non-subtype B infections [16]. Env immunogens that are capable of triggering a bnAb response may thus harbor distinct phenotypic features that facilitate bnAb precursor binding or mAb maturation. This could include a multitude of features that influence epitope exposure such as the degree of shielding, Env stability, the density of the trimeric spike on virions or specific conformation traits that affect the exposure of the target epitope.

bnAb evolution has been shown to benefit from viral diversity [13,16–28,30,39]. Viral populations which have evolved to high diversity or harbor high diversity due to superinfection, as in the case of patient CAP256, represent a mixture of Env immunogens that undoubtedly will widely differ in phenotype. It is feasible that the necessity to simultaneously react with highly differential epitopes, e.g. open (neutralization sensitive, epitope exposing) and closed (neutralization resistant, epitope largely hidden) immunogens, may steer the immune system into recognizing a broad variety of Env conformations. Intriguingly, such a dichotomy in sensitivity was present in CAP256 during VRC26 development with the highly VRC26 resistant PI strain and the VRC26 sensitive SU strain co-circulating eventually resulting in the evolution of broad VRC26 variants that harbor activity also against the PI strain [33,41] (Figs 1 and 2).

Other phenotypic aspects and characteristics of the virus-Ab interplay are also likely to be important. Slow escape from the bnAb lineage may be beneficial by prolonging virus-bnAb evolution and allowing breadth to develop [63]. Comparatively higher initial resistance to neutralization or a higher tolerance to mutations in Env could thereby prove beneficial.

Likewise, replication properties may be important. As escape from CAP256 highlights, the kinetics of the entry process may influence neutralization efficacy by altering the window of action for neutralization. A differential capacity to replicate as free virus or by cell-cell transmission has been recognized to affect neutralization activity, with cell-cell transmission often less effectively inhibited by nAbs. This results in a higher propensity to select for resistance mutations in cell-cell transmission [47,49–52,54,63,64]. As we show here, VRC26 is one of the few antibodies that retains substantial activity against cell-associated virus. This was particularly evident for virus strains for which VRC26 has a comparatively lower activity in free virus transmission (Fig 2E) [52]. The fact that with increasing VRC26 resistance the transmission mode appears to have less influence on nAb efficacy is intriguing. This could imply that the Env of evolved strains adopts an Env conformation in the unliganded stage that resembles Env conformations that are relevant during cell-cell transmission. By investigating the phenotypic properties of autologous virus strains which circulated prior to and during the evolution of the bnAb CAP256-VRC26 lineage we show that the capacity of VRC26 to maintain activity during cell-cell transmission is shared by other V2 apex bnAbs, suggesting that their common mechanism of action contributes to this. What constitutes the differences for certain bnAbs but not others in the context of free and cell-cell transmission will be important to resolve in forthcoming studies. Multiple scenarios may apply, and dissecting whether a reduced capacity results from an inability to recognize Env on recently budded, immature viruses or the CD4 bound Env will be of particular interest.

The capacity of VRC26 to act before and after CD4 engagement provides a first clue to how VRC26 is equally effective in cell-cell transmission [52] as it allows the bnAb to be active over a longer time window during the entry process. Considering that attachment to CD4 in cell-cell transmission is rapid, the capacity to block entry after CD4 binding is likely a benefit for cell-cell neutralization activity [52].

The first wave of VRC26 escape variants we investigated here thus hints at an intriguing phenotypic plasticity in Env functionality. While the virus managed to maintain its Env replication competence, these changes seem to have come at entry fitness costs in the initial phase of resistance adaptation to VRC26. Both, a loss in replicative fitness of the initial escape variants and the sustained sensitivity to VRC26 during cell-cell-spread may have aided the evolution of VRC26 breadth by reducing virus progeny and thus slowing formation of escape variants. Prolonged exposure until full escape is reached will favor the long-term circulation of virus variants with an intact VRC26 epitope, thereby increasing the chances for the bnAb response to mature. Indeed, slow viral escape has been reported in several bnAb lineages [32,34,63,65] though the mechanism for this has not been clear as passive administration of bnAbs results in rapid viral escape [66–70].

In summary, our findings illustrate the phenotypic plasticity of the HIV-1 Env in evading bnAb pressure. As exemplified by VRC26, alterations in phenotypic traits that emerge in response to a bnAb response can provide insights into functional consequences of viral escape and potentially may highlight differential Env conformations that should be considered when selecting and designing Env immunogens. Sequential immunization protocols to mature bnAb responses by vaccination may be beneficial if combinations of Env variants with differential phenotypic patterns and bnAb sensitivity, as we describe here for CAP256, are included.

## Materials and methods

### Ethics statement

A cryo-preserved plasma sample from week 145 p.i. of the CAP256 patient for the analyses of the current study was provided by the repository of the CAPRISA 002 Acute Infection study, Durban, South Africa [71]. The CAPRISA 002 study was reviewed and approved by the research ethics committees of the University of KwaZulu-Natal (E013/04), the University of Cape Town (025/2004) and the University of the Witwatersrand (MM040202).

### Antibodies

CAP256 VRC26 bnAbs and expression plasmids have been described previously [33,41]. We thank D. Burton, J. Mascola, M. Nussenzweig and M. Connors for providing antibodies and antibody expression plasmids for this study either directly or via the NIH AIDS Research and Reference Reagent Program (NIH ARP). All antibodies were expressed in FreeStyle 293-F cells and purified on protein G affinity and size exclusion chromatography columns as described [72].

### Inhibitors

The CD4-directed DARPin 55.2 was produced as described [61], the fusion inhibitor T-20 [62] was purchased from Roche Pharmaceuticals.

### Cells

293-T cells (obtained from the American Type Culture Collection (ATCC)) and TZM-bl cells ([73]; obtained from the National Institute of Health AIDS Reagent Program) were maintained in DMEM with 10% heat inactivated FCS and 1% Penicillin/Streptomycin. FreeStyle 293-F cells were purchased from Thermo Fisher Scientific and maintained according to the manufacturers instructions. A3.01-CCR5 cells were previously generated [47] and were cultivated in RPMI with 10% heat inactivated FCS and 1% Penicillin/Streptomycin.

### Viruses

Envelope (Env) genes of patient CAP256 were previously cloned as described [33,34,42] and GenBank accession numbers are summarized in S1 Table. We only had the capacity to include a selection of available Env clones in the current study, to allow an assessment of all variants in the same assay runs to restrict assay variability. We primarily focused on multiple Env variants from key time points prior to and during the emergence of the VRC26 lineage, when the autologous viruses were still partially sensitive to VRC26 [34] as the interaction with VRC26 was a main aim of our study. The 34wk and 48wk time points thus allowed a clear focus on VRC26-mediated pressure. We did not include strains beyond wk176 as these viruses are resistant to VRC26. For certain complex and labor intensive analyses (e.g. kinetics assay and pre-post attachment assay), only a restricted virus panel was assessed to still allow processing of all Env variants in the same assay run. As the emphasis of our study was on the SU viruses, these were preferentially included. Amongst PI viruses, we preferentially included later viruses over earlier (23wk^PI^ and 30wk^PI^) variants as phenotypic evolution in later clones which experienced VRC26 potentially are of higher interest in the context of the current study.

For the production of single-round replicating cell-free pseudovirus stocks, 293-T cells were transfected with the luciferase reporter HIV-1 pseudotyped vector pNLlucAM [74] (a gift from A. Marozsan and J. P. Moore) and the respective Env expression plasmids as described [75]. Infectivity of reporter viruses was quantified by titration of virus containing supernatants on 1*10^4^ TZM-bl or 5*10^4^ A3.01-CCR5 in a 1:4 ratio starting from 100 μl virus solution/well in the presence of 10 μg/ml diethylaminoethyl (DEAE, Amersham Biosciences, Connecticut, USA). Infection of target cells was assessed by measuring the firefly luciferase activity from the lysed cells using firefly luciferase substrate (Promega, Madison Wisconsin, USA). Emitted RLU were quantified on a Dynex MLX luminometer (Dynex Technologies Inc., Chantilly, Virginia, USA).

Infection via the cell-cell-route is described below. As the A3.01-CCR5 cell-cell transmission assay relies on the omission of polycations to exclude free virus spread, all CAP256 Env isolates used in the current study were confirmed to require polycations for free virus entry (S7 Fig).

### Comparing CAP256 Env infectivity in free virus and cell-cell transmission

To assess viral infectivity in free virus cell-cell transmission, we employed recently described assay formats utilizing A3.01-CCR5 cells as target cells and either cell-free virus or 293-T transfected cells as source of virus [52]. In both systems, a reporter virus backbone lacking *env* and an *env* expression vector are co-transfected to generate Env pseudoviruses or Env pseudovirus expression cells, respectively. An essential difference in the free virus and the cell-cell transmission assay is the virus backbone used. The free virus set up uses the conventional NL4-3 based pNLlucAM luciferase reporter Env pseudotyping backbone [74]. The cell-cell transmission assay utilizes a NL4-3 derived pseudotyping HIV-1 backbone with an intron-regulated Gaussia luciferase LTR-reporter construct called inGluc (kind gift from Dr. M Johnson [76–78]). The reverse orientation of the reporter and the intron allow luciferase expression only after correct splicing, packaging into viral particles and infection of A3.01-CCR5 target cells. Free virus infection in the cell-cell transmission set up is restricted by the omission of DEAE in the infection medium as described previously [47].

To measure infectivity in free virus and cell-cell transmission, 5*10^4^ 293-T cells per 24-well were transfected with Env and NLinGluc backbone for cell-cell transmission or pNLlucAM backbone plasmids for free virus transmission in a 1:3 ratio, using polyethyleneimine (PEI) as transfection reagent. To test cell-cell infectivity, 293-T cells were transfected with the inGluc reporter and an Env plasmid of choice. After 6 h incubation, cells were detached and 5*10^3^ transfected cells /100 μl RPMI medium seeded per 96 well. A3.01-CCR5 target cells (1.5*10^4^ /100 μl RPMI medium) were added to each well. After 65 h of incubation at 37°C, Gaussia luciferase activity in the supernatant was quantified using the Renilla Luciferase Assay System (Promega, Madison Wisconsin, USA) according to the manufacturer’s instructions.

To test free virus infectivity, 293-T cells transfected with the pNLlucAM luciferase reporter and an Env expression plasmid were incubated for a total of 65 h, and the transfection medium exchanged after 8 h. After 65 h, the supernatant was collected, briefly centrifuged at maximum speed and frozen for 24 h to prevent carry-over of transfected cells. 100 μl of a 1:2 virus dilution with DMEM medium were seeded into 96 well plates in duplicates or triplicates and 5*10^4^ A3.01-CCR5 target cells in 100 μl per 96 well in the presence of 10 μg/ml DEAE were added. After 65 h incubation at 37°C, infection was assessed by luciferase production after cell lysis and addition of firefly luciferase substrate (Promega, Madison, Wisconsin, USA).

### Neutralization of cell-free Env-pseudotyped viruses on A3.01-CCR5

Free virus inhibition by bnAbs and plasma was assessed on A3.01-CCR5 cells using Env-pseudotyped NLlucAM reporter viruses as described [52]. Briefly, a virus input of around 10,000 relative light units (RLU) per 96 well in absence of inhibitors was pre-incubated with the respective bnAbs or patient plasma for 1 h at 37°C. 5*10^4^ A3.01-CCR5 target cells per 96 well in the presence of 10 μg/ml DEAE were added and incubated for 65 h at 37°C. Infection was assessed by firefly luciferase production as described above. The inhibitor concentrations or plasma dilutions causing 50% reduction in viral infectivity (50% inhibitory concentration; IC50 or 50% neutralizing titers, NT50) were calculated by fitting pooled data from two to four independent experiments to sigmoid dose response curves (variable slope) using GraphPad Prism. If 50% inhibition was not achieved at the highest bnAb concentration or lowest plasma dilution, a greater-than value was recorded.

### Measuring neutralization activity during cell-cell transmission of 293-T to A3.01-CCR5 cells

For measuring neutralization of cell-cell transmission, 293-T cells were transfected with Env and NLinGluc plasmids in a 1:3 ratio. 6 h post transfection, 5*10^3^ transfected cells were seeded in 50 μl per 96 well and serial dilutions of bnAbs or patient plasma in 50 μl per 96 well were added. After 1 h incubation at 37°C, 1.5*10^4^ A3.01-CCR5 target cells in 100 μl RPMI medium were added for 65 h at 37°C. Gaussia luciferase activity in the supernatant was quantified using the Renilla Luciferase Assay System (Promega, Madison Wisconsin, USA) according to the manufacturer’s instructions. Neutralization data were analyzed with GraphPad Prism as described above.

The change in neutralization activity for cell-cell compared to free virus transmission (fold change IC50) was calculated as the ratio of the IC50 of neutralization during cell-cell and free virus transmission. If for only one of the pathways, the virus was resistant to a particular bnAb, the IC50 was nominally set to a value of two times the highest ineffective bnAb-concentration tested for that pathway.

### Measuring viral entry kinetics

Entry kinetics were assessed using an inhibitor time of addition set up as recently described [59,60] (Fig 6A). The essence of the assay is a synchronized infection of free virus that is blocked at distinct time points by inhibitors interfering with CD4 attachment (the CD4 blocking agent DARPin 55.2 [61]) or fusion (T-20 [62]).

Briefly, 6*10^3^ TZM-bl cells in 60 μl DMEM medium per 384-wells were seeded and incubated for 24 h at 37°C. Cells were then shortly cooled at 4°C before removing the medium and adding HIV-1 pseudovirus stocks yielding 50’000 RLU in 60 μl DMEM medium with 10 μg/ml DEAE-Dextran at 4°C per 384-well. Virus binding to the cells was synchronized by spinoculation of the plates for 30 min at 2095 g and 4°C. Supernatants including unbound viruses were removed, 37°C warm DMEM was added to start the infection and plates were incubated at 37°C. At indicated time points, the infection was stopped by the addition of inhibitors of CD4 binding (1 μM DARPin 55.2) or fusion (50 μg/ml T-20). The chosen concentration of each of the inhibitors exceeded the 100% inhibitory concentrations, which had been determined for the individual virus strains. After 48 h incubation at 37°C, virus infection was quantified by measuring the Gaussia luciferase activity in the supernatant as described. To allow comparison of virus infectivity across independent experiments, infectivity after 120 min was set to 100% and virus infectivity in all other wells were expressed in relation to this. The time to reach 50% of infection (half-maximal entry times, t_1/2_) was used as a surrogate for timing of CD4 receptor binding or fusion. For each Env, each inhibitor and each replicate, a general kinetic equation

(A-D)/(1+/(x/C)^B)+D

was fitted to the time series of the data points and a t_1/2_ value was estimated from the fitted equation. If the least-squares approximation used to fit the kinetic equation did not converge, a straight line was instead used to estimate t_1/2._ To deal with irregularities of the data, this line connects the data point left of the first point with >50% relative infectivity and the point right of the last point with <50% relative infectivity. The reported t_1/2_ value for each Env and each inhibitor is the mean t_1/2_ value across all replicates. To compare the time intervals between three different stages of the entry process (synchronized start, CD4 binding, fusion) among different Env, Mann-Whitney tests were performed. Only Env of the same type (PI-like or SU-like) were compared.

Infection time courses for both inhibitors were generated and the mean half-maximal time (mean t_1/2_) required for viruses to progress with the entry process beyond these two steps were calculated from the fitted curves (Fig 6B, S4 Fig). Time intervals between three different stages of the entry process (CD4 attachment, progress from CD4 to fusion, and fusion) among either the PI-like or SU-like Env were compared (S5 Fig).

### Measuring the pre- and post-CD4 attachment neutralization activity

The neutralization capacity of bnAbs at pre- and post-attachment of NLlucAM reporter viruses to A3.01-CCR5 target cells was assessed as described [47,52]

Briefly, total neutralization activity at the pre- and post-attachment stage was measured by pre-incubating NLlucAM reporter viruses yielding around 10’000 RLU per 96 well with the respective bnAbs for 1 h at 37°C. The virus-bnAb mix was then spinoculated onto 1*10^5^ A3.01-CCR5 target cells in RPMI with 50 μM Hepes and 10 μg/ml DEAE per 96 well for 2 h at 1200 g and 23°C. The reaction was transferred to 37°C and incubated for 65 h.

To assess pre-attachment neutralization activity, samples were additionally washed after the spinoculation step to remove unbound viruses and inhibitors.

To assess inhibitory capacity at the post-CD4 attachment step, NLlucAM reporter viruses were first spinoculated onto the A3.01-CCR5 target cells and then bnAbs were added for 1 h incubation at 23°C before rising the temperature to 37°C for 65 h.

Infectivity was determined by firefly luciferase reporter production from the lysed cells as described. Samples measuring the total inhibition activity were set to 100% and pre- and post-attachment samples were expressed relative to the total activity.

### Phylogenetic analysis of the envelope genes

We reconstructed a phylogeny based on the Env sequences of the 17 CAP256 clones included in the present study. The CAP256 Env sequences [34] were aligned to the HXB2 genome using AliView [79]. The phylogenetic analysis was performed employing the sampled ancestor model [80] in BEAST2 [81]. The maximum credibility tree was constructed with TreeAnnotator and the phylogeny displayed with FigTree. The resulting summary of the posterior distribution of phylogenies is shown in S2A Fig. The entire posterior distribution of phylogenetic trees is displayed with DensiTree [82] in S2B Fig. The xml-file of the BEAST2 analysis is summarized in S1 Dataset.

### Statistical analysis

Correlation analyses according to Spearman using the untransformed data sets were performed in GraphPad Prism. Unmatched groups were compared using the nonparametric Mann-Whitney test in GraphPad Prism.

## Supporting information

S1 Fig CAP256-VRC26 bnAbs maintain a high neutralization activity against cell-cell transmission of autologous viruses. Comparison of VRC26 bnAbs according to 50% inhibitory concentrations (IC50 in μg/ml) against free virus and cell-cell transmission and fold change IC50^cell-cell^ /IC50^free virus^. **A:** Neutralization activity is shown for VRC26 bnAbs sorted by heterologous breadth, determined on a panel of 46 heterologous viruses (S2 Table [41]). **B:** Spearman correlation on untransformed data sets of VRC26 bnAb neutralization activity and their heterologous breadth for free virus and cell-cell transmission. No significant interrelation was detected (Spearman correlation, R: -0.3585, p = 0.2508 and R: -0.3199, p = 0.3085 respectively). **C:** Neutralization activity is shown for VRC26 bnAbs sorted by bnAb maturation, defined by the proportion of amino acid changes in the heavy chain from the unmutated common ancestor (UCA; [41]. **D:** Spearman correlation on untransformed data sets of VRC26 bnAb neutralization activity and the proportion of amino acid changes in the heavy chain of the UCA for free virus and cell-cell transmission. No significant interrelation was detected (Spearman correlation, R: 0.1056, p = 0.7480 and R: 0.007042, p = 0.9916 respectively). **A+C:** Black lines show the median IC50 or fold change IC50 of all sensitive combinations for each bnAb. PI-like, *SU-like VRC26 sensitive* and *SU-like VRC26 early escape* viruses are marked in red, light blue and dark blue respectively.
(PDF)

S2 Fig Time-resolved phylogeny of all viral sequences isolated from CAP256. **A:** The CAP256 phylogeny represents the maximum credibility tree of a BEAST2 analysis and is based on 17 CAP256 Env variants listed in S1 Table. Each node is provided with the posterior probability of this node and the 95% HPD (highest posterior density) interval. **B:** Representation of the trees visited and accepted by the Markov Chain Monte Carlo (MCMC) algorithm of the BEAST2 phylogenetic analysis. The low posterior probabilities at many branching events **(A)** and the distribution of trees **(B)** show that the phylogenetic tree cannot be unambiguously determined due to the previously documented recombination among the primary infecting PI and SU strains [34,42]. The time line is orientated backwards in time with week 0 as the time point of the last sample date included.
(PDF)

S3 Fig Activity of autologous plasma against cell-cell transmission of CAP256 viruses is strongly driven by VRC26 bnAb activity. Scatter blots for the correlation analysis presented in Fig 5C and 5D. **A:** Interrelations of neutralizing titers for plasma and IC50s for bnAb neutralization for PI-like and SU-like viruses during free virus and cell-cell transmission. **B:** Interrelations of virus infectivity in free virus and cell-cell transmission, IC50s and neutralizing titers (NT50) for SU-like viruses. **A+B:** Spearman correlations on untransformed data sets were used, R and p values are indicated. Significant correlations are marked in red. N.s. denotes no significance.
(PDF)

S4 Fig Entry kinetics of CAP256 viruses. Entry kinetics infection curves were obtained by the synchronized infection of TZM-bl cells and the addition of CD4-attachment inhibitor DARPin 55.2 or fusion inhibitor T-20 at indicated time points to block infection. Infection curves were fitted using data points from individual experiments and the mean half-maximal entry times (t_1/2_) were determined from two to four independent experiments. The fits for one representative experiment are shown.
(PDF)

S5 Fig Virus evolution alters the entry kinetics of CAP256 viruses. Heat maps showing the statistical differences for t_1/2_ to CD4 attachment, fusion and the time between CD4 attachment and fusion. Statistical significance was determined with Mann-Whitney tests and shades of green indicate p values (dark green denotes a low p value/strong difference).
(PDF)

S6 Fig A decreased sensitivity to neutralization by the CAP256-VRC26 bnAbs is associated with viral fitness losses. Scatter blots for the correlation analysis presented in Fig 6C. Interrelations of IC50s (in μg/ml) for VRC26 bnAb neutralization, viral infectivity and mean half-maximal time (t_1/2_) to CD4-attachment, fusion and CD4 attachment to fusion were determined separately for SU-like (left) and PI-like (right) viruses during free virus and cell-cell transmission. Spearman correlations on untransformed data sets were used, R and p values are indicated. Significant correlations are marked in red. N.s. denotes no significance.
(PDF)

S7 Fig The DEAE omission system to restrict free virus spread in cell-cell analyses is applicable for all autologous CAP256 viruses. CAP256 NLlucAM reporter pseudoviruses were titrated on A3.01-CCR5 cells in 96 well plates in presence (black) or absence (gray) of 10 μg/ml diethylaminoethyl (DEAE). Firefly luciferase reporter activity was measured from the lysed cells. The maximum virus input used for free virus neutralization assays is indicated (dashed line).
(PDF)

S1 Table Longitudinal CAP256 Env panel. Overview of CAPR256 Env variants used in the current study.
(PDF)

S2 Table CAP256-VRC26 bnAb characteristics and autologous free virus neutralization. Overview of CAP256-VRC26 bnAbs used in the current study.
(PDF)

S3 Table Comparison heterologous bnAb neutralization of VRC26 sensitive and early escape SU-like viruses. Comparison (Mann-Whitney test) of sensitivity of VRC26 sensitive and early escape SU-like viruses to heterologous bnAbs directed to different epitopes.
(PDF)

S1 Dataset CAP256 Env sequence analysis. xml-file of BEAST2 analysis.
(TXT)
