# Supplementary material for: Phenotypic deficits in the HIV-1 envelope are associated with the maturation of a V2-directed broadly neutralizing antibody lineage
Source: PLoS Pathog. 2018 Jan 25;14(1):e1006825. doi: 10.1371/journal.ppat.1006825 (PMC5806907; doi:10.1371/journal.ppat.1006825)
Supplement: S1 Fig — Comparison of VRC26 bnAbs according to 50% inhibitory concentrations (IC50 in μg/ml) against free virus and cell-cell transmission and fold change IC50cell-cell /IC50free virus. A: Neutralization activity is shown for VRC26 bnAbs sorted by heterologous breadth, determined on a panel of 46 heterologous viruses (S2 Table [41]). B: Spearman correlation on untransformed data sets of VRC26 bnAb neutralization activity and their heterologous breadth for free virus and cell-cell transmission. No significant interrelation was detected (Spearman correlation, R: -0.3585, p = 0.2508 and R: -0.3199, p = 0.3085 respectively). C: Neutralization activity is shown for VRC26 bnAbs sorted by bnAb maturation, defined by the proportion of amino acid changes in the heavy chain from the unmutated common ancestor (UCA; [41]. D: Spearman correlation on untransformed data sets of VRC26 bnAb neutralization activity and the proportion of amino acid changes in the heavy chain of the UCA for free virus and cell-cell transmission. No significant interrelation was detected (Spearman correlation, R: 0.1056, p = 0.7480 and R: 0.007042, p = 0.9916 respectively). A+C: Black lines show the median IC50 or fold change IC50 of all sensitive combinations for each bnAb. PI-like, SU-like VRC26 sensitive and SU-like VRC26 early escape viruses are marked in red, light blue and dark blue respectively. (PDF) [file ppat.1006825.s001.pdf]

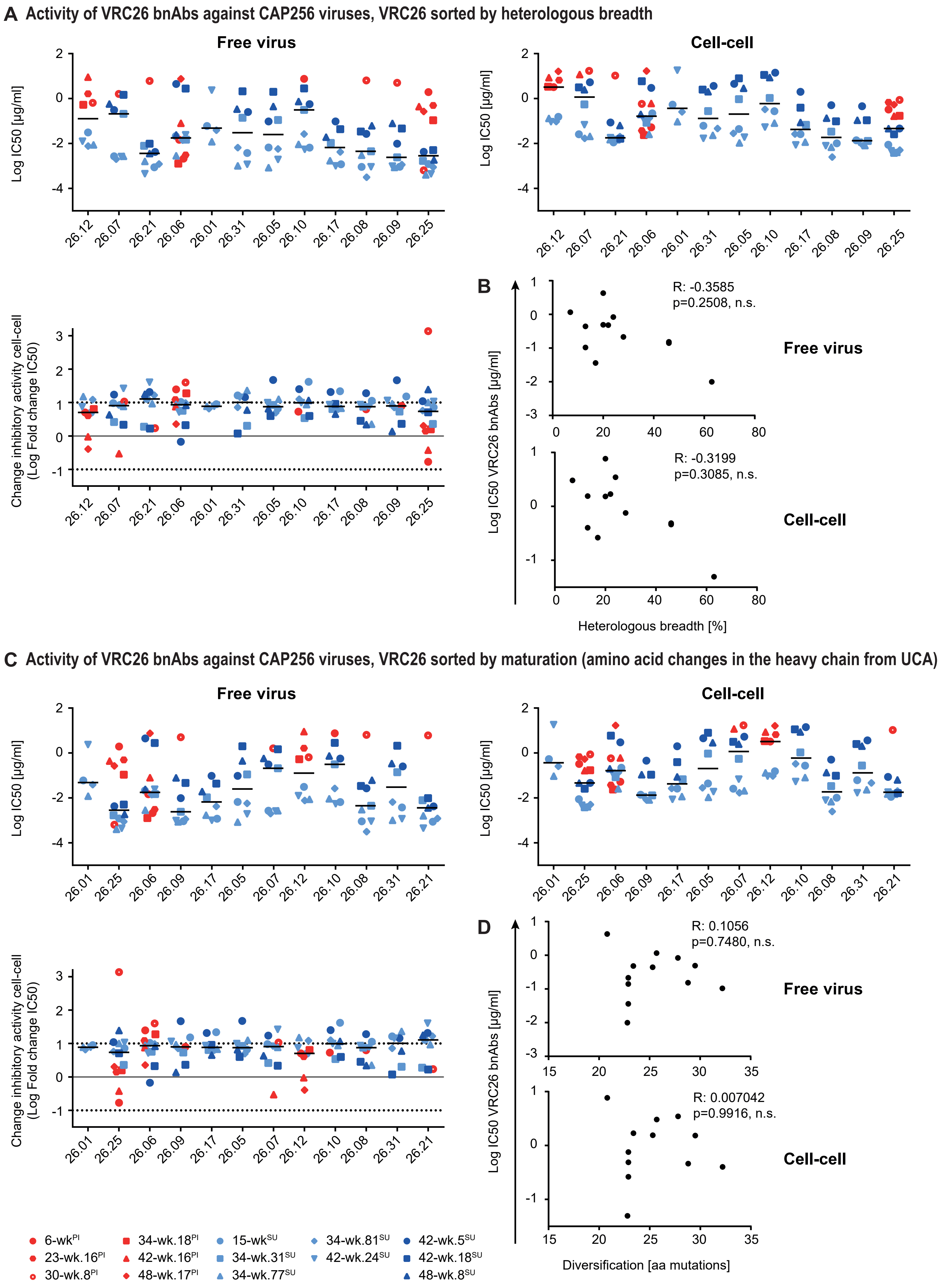
A Activity of VRC26 bnAbs against CAP256 viruses, VRC26 sorted by heterologous breadth
Free virus
Cell-cell
Log IC50 [µg/ml]
26.12 26.07 26.21 26.06 26.01 26.31 26.05 26.10 26.17 26.08 26.09 26.25
Change inhibitory activity cell-cell (Log Fold change IC50)
B
Log IC50 VRC26 bnAbs [µg/ml]
R: -0.3585
p=0.2508, n.s.
Free virus
R: -0.3199
p=0.3085, n.s.
Cell-cell
Heterologous breadth [%]
C Activity of VRC26 bnAbs against CAP256 viruses, VRC26 sorted by maturation (amino acid changes in the heavy chain from UCA)
Free virus
Cell-cell
Log IC50 [µg/ml]
26.01 26.25 26.06 26.09 26.17 26.05 26.07 26.12 26.10 26.08 26.31 26.21
Change inhibitory activity cell-cell (Log Fold change IC50)
D
Log IC50 VRC26 bnAbs [µg/ml]
R: 0.1056
p=0.7480, n.s.
Free virus
R: 0.007042
p=0.9916, n.s.
Cell-cell
Diversification [aa mutations]
6-wk^PI
23-wk.16^PI
30-wk.8^PI
34-wk.18^PI
42-wk.16^PI
48-wk.17^PI
15-wk^SU
34-wk.31^SU
34-wk.77^SU
34-wk.81^SU
42-wk.24^SU
42-wk.5^SU
42-wk.18^SU
48-wk.8^SU
