# Supplementary figures and images for: Phenotypic deficits in the HIV-1 envelope are associated with the maturation of a V2-directed broadly neutralizing antibody lineage
Source: PLoS Pathog. 2018 Jan 25;14(1):e1006825. doi: 10.1371/journal.ppat.1006825 (PMC5806907; doi:10.1371/journal.ppat.1006825)

**A**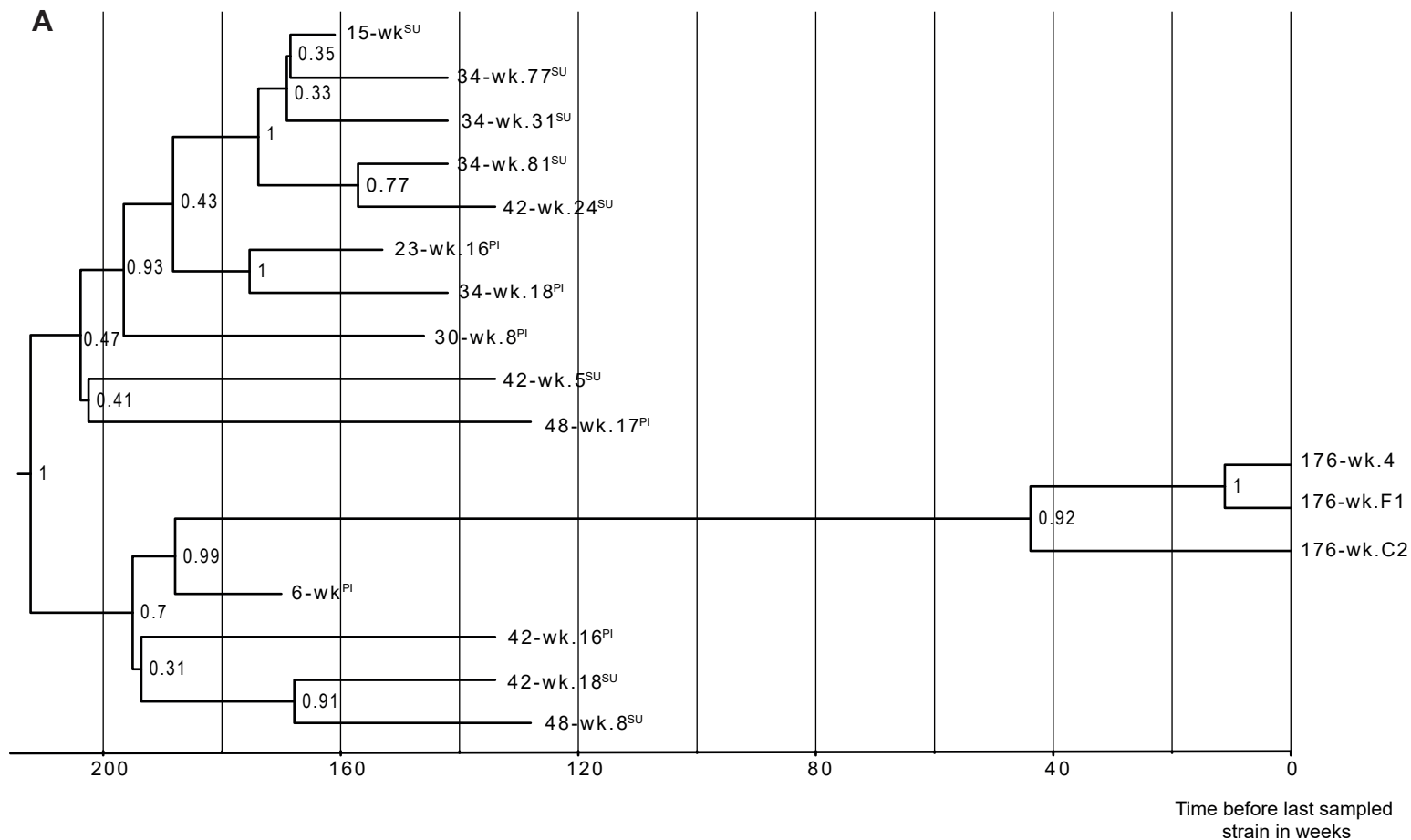**B**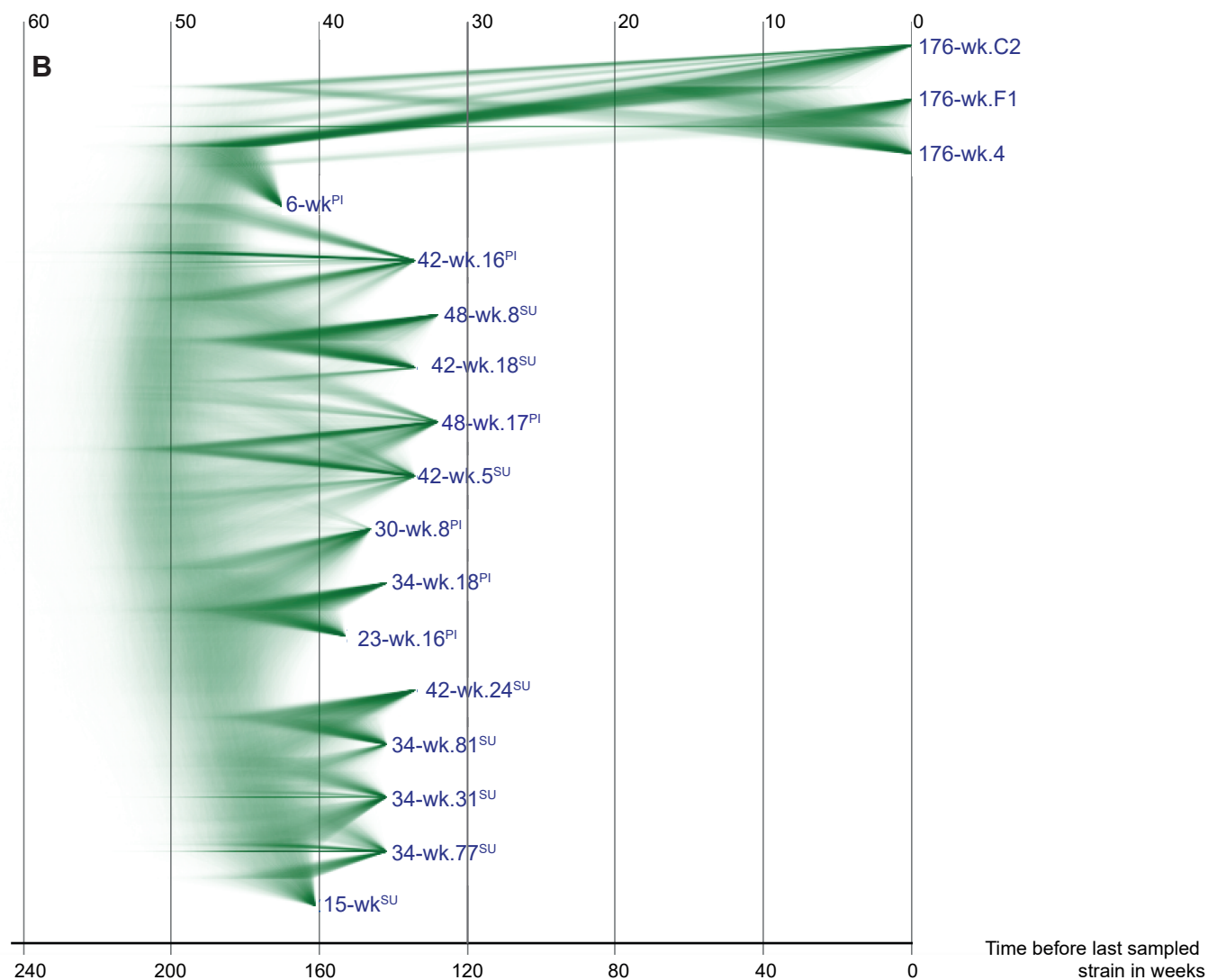

Supplement: S2 Fig — A: The CAP256 phylogeny represents the maximum credibility tree of a BEAST2 analysis and is based on 17 CAP256 Env variants listed in S1 Table. Each node is provided with the posterior probability of this node and the 95% HPD (highest posterior density) interval. B: Representation of the trees visited and accepted by the Markov Chain Monte Carlo (MCMC) algorithm of the BEAST2 phylogenetic analysis. The low posterior probabilities at many branching events (A) and the distribution of trees (B) show that the phylogenetic tree cannot be unambiguously determined due to the previously documented recombination among the primary infecting PI and SU strains [34,42]. The time line is orientated backwards in time with week 0 as the time point of the last sample date included. (PDF) [file ppat.1006825.s002.pdf]

**A**

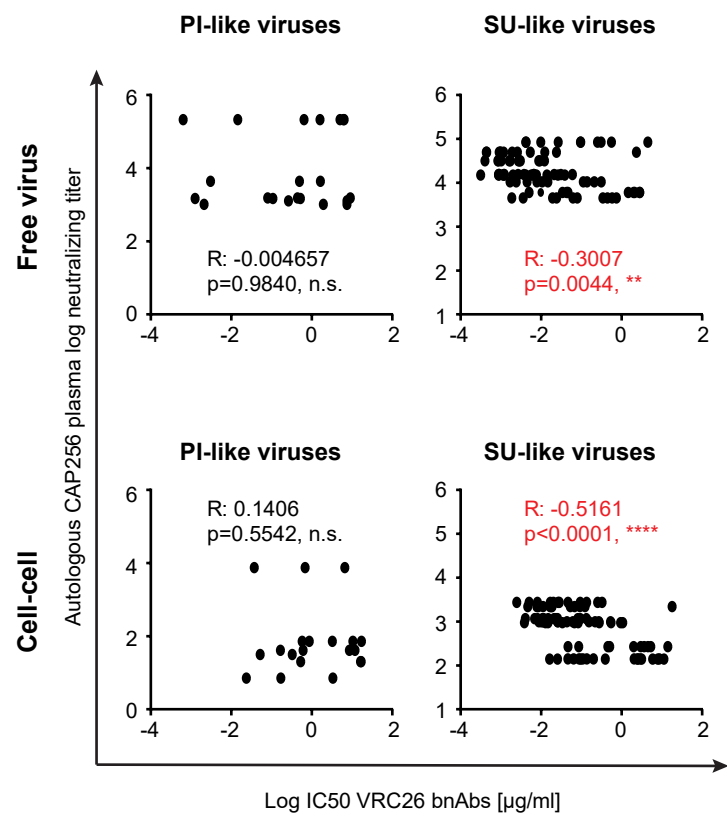

**B**

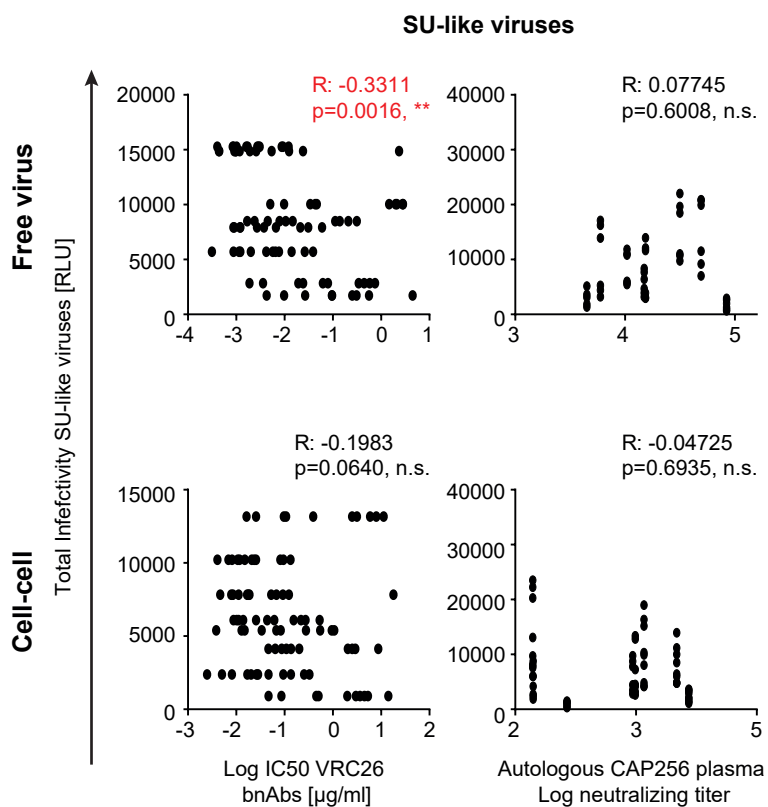

Supplement: S3 Fig — Scatter blots for the correlation analysis presented in Fig 5C and 5D. A: Interrelations of neutralizing titers for plasma and IC50s for bnAb neutralization for PI-like and SU-like viruses during free virus and cell-cell transmission. B: Interrelations of virus infectivity in free virus and cell-cell transmission, IC50s and neutralizing titers (NT50) for SU-like viruses. A+B: Spearman correlations on untransformed data sets were used, R and p values are indicated. Significant correlations are marked in red. N.s. denotes no significance. (PDF) [file ppat.1006825.s003.pdf]

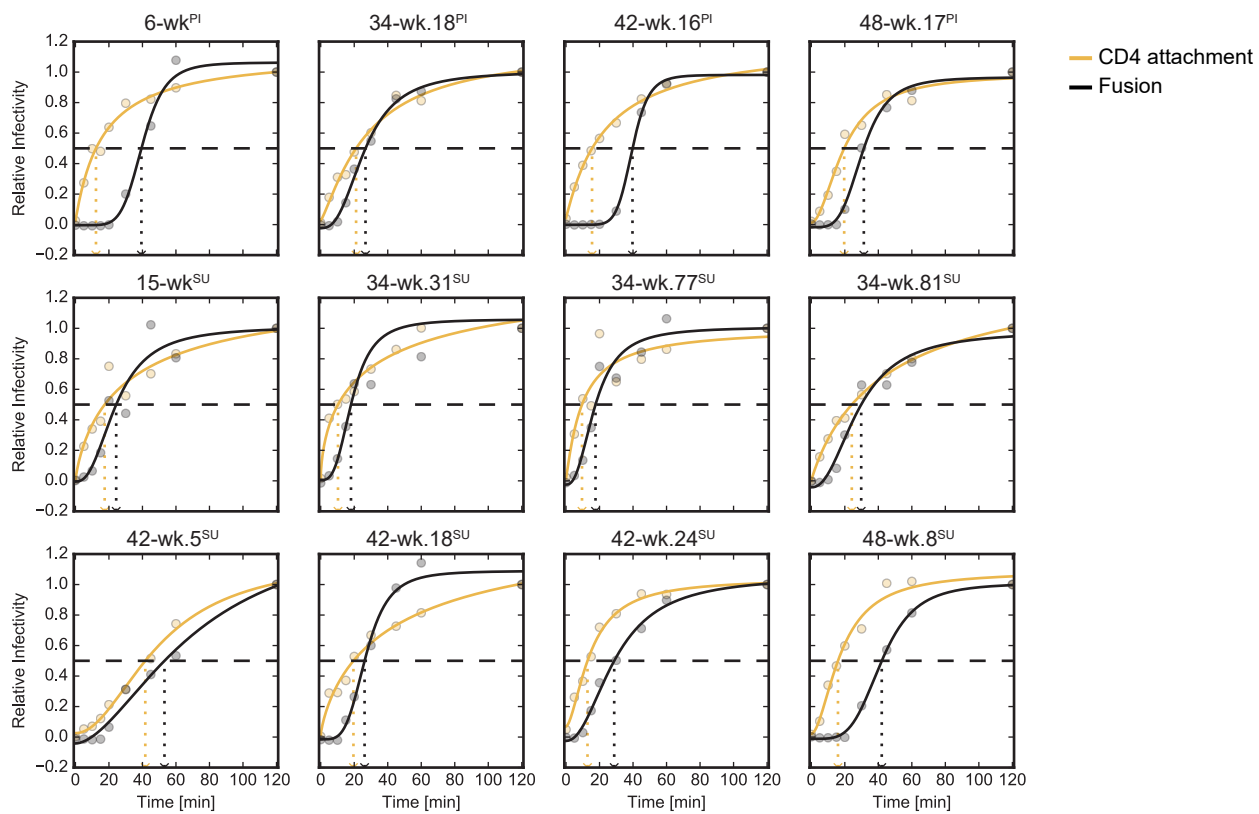

Supplement: S4 Fig — Entry kinetics infection curves were obtained by the synchronized infection of TZM-bl cells and the addition of CD4-attachment inhibitor DARPin 55.2 or fusion inhibitor T-20 at indicated time points to block infection. Infection curves were fitted using data points from individual experiments and the mean half-maximal entry times (t1/2) were determined from two to four independent experiments. The fits for one representative experiment are shown. (PDF) [file ppat.1006825.s004.pdf]

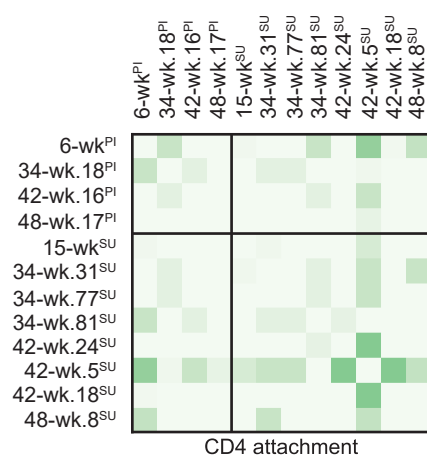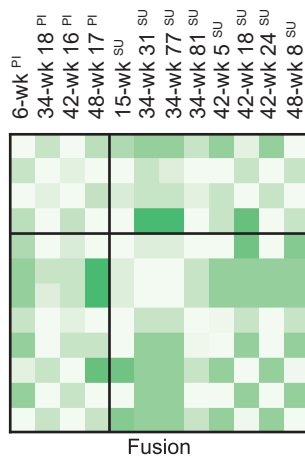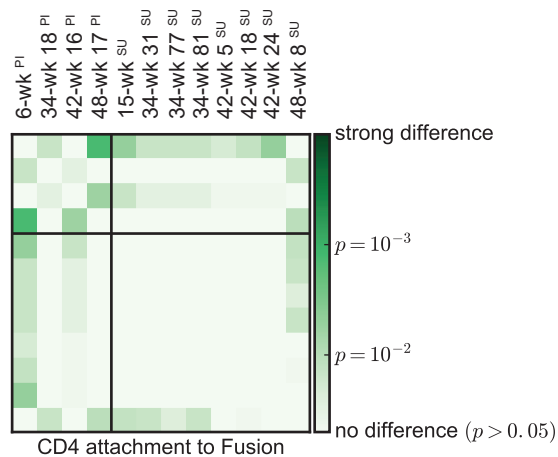

Supplement: S5 Fig — Heat maps showing the statistical differences for t1/2 to CD4 attachment, fusion and the time between CD4 attachment and fusion. Statistical significance was determined with Mann-Whitney tests and shades of green indicate p values (dark green denotes a low p value/strong difference). (PDF) [file ppat.1006825.s005.pdf]

Free virus

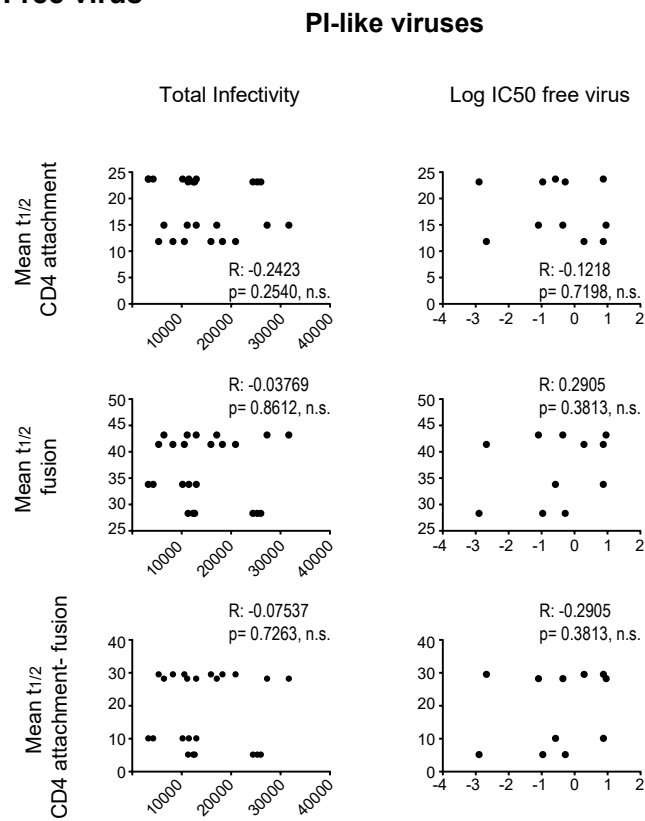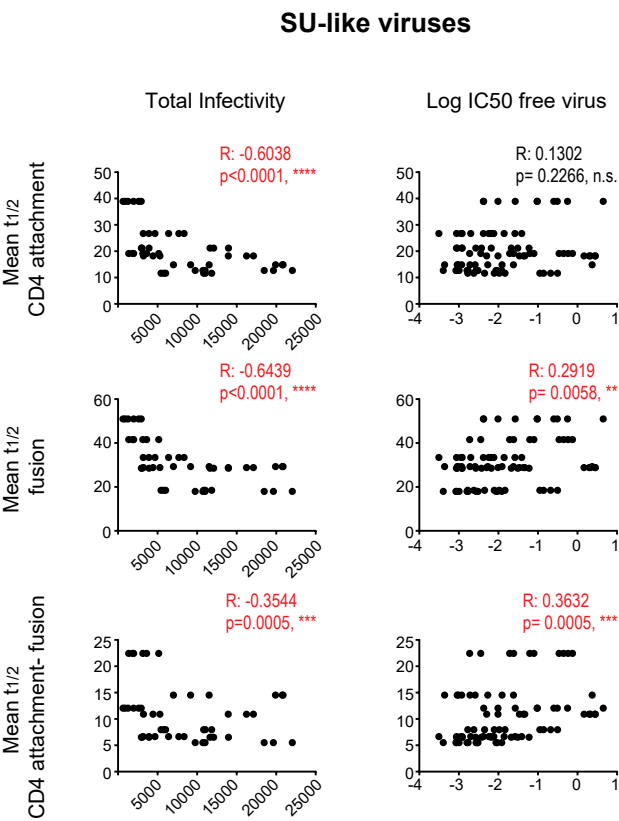

Cell-cell

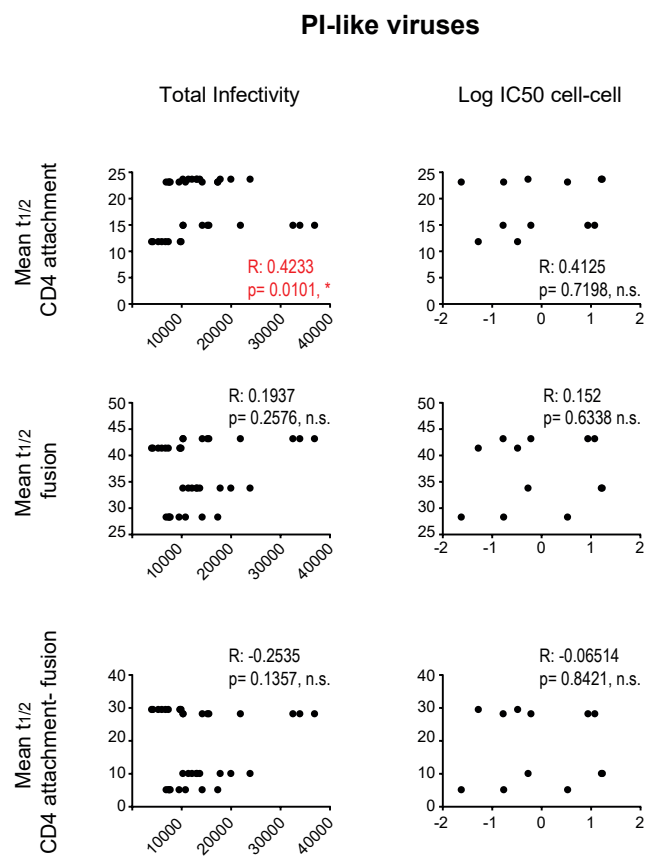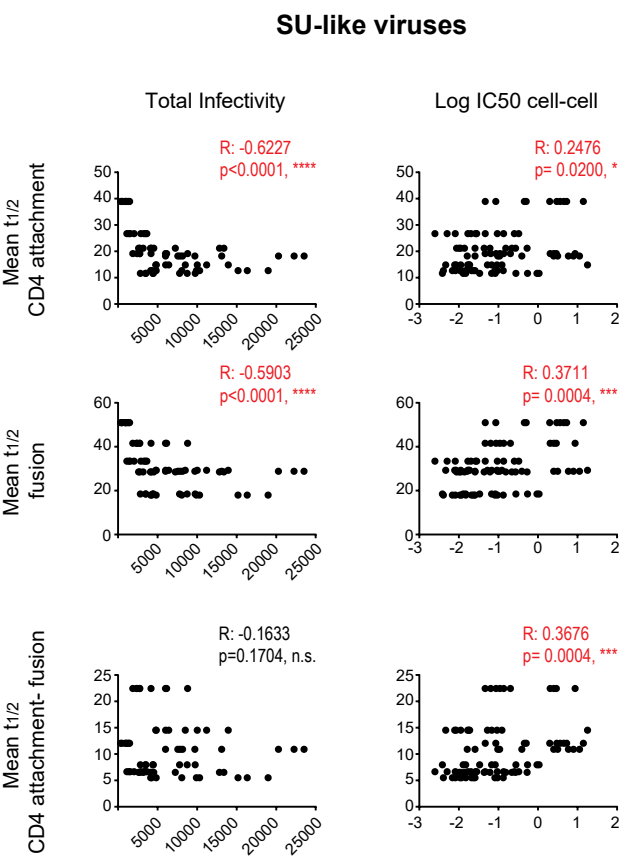

Supplement: S6 Fig — Scatter blots for the correlation analysis presented in Fig 6C. Interrelations of IC50s (in μg/ml) for VRC26 bnAb neutralization, viral infectivity and mean half-maximal time (t1/2) to CD4-attachment, fusion and CD4 attachment to fusion were determined separately for SU-like (left) and PI-like (right) viruses during free virus and cell-cell transmission. Spearman correlations on untransformed data sets were used, R and p values are indicated. Significant correlations are marked in red. N.s. denotes no significance. (PDF) [file ppat.1006825.s006.pdf]

Total infectivity [RLU]

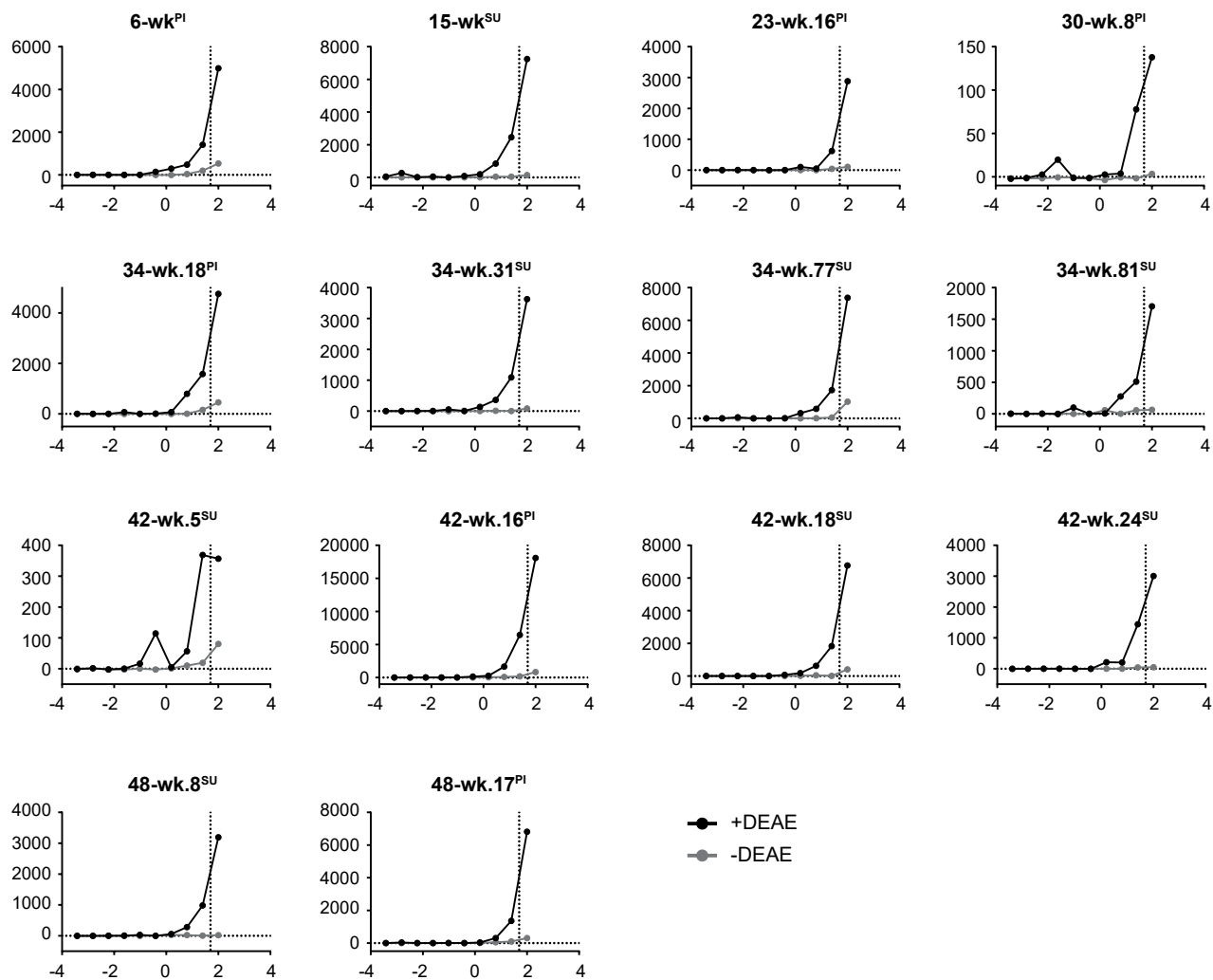

Virus input [ $\mu\text{l}$ ]

Supplement: S7 Fig — CAP256 NLlucAM reporter pseudoviruses were titrated on A3.01-CCR5 cells in 96 well plates in presence (black) or absence (gray) of 10 μg/ml diethylaminoethyl (DEAE). Firefly luciferase reporter activity was measured from the lysed cells. The maximum virus input used for free virus neutralization assays is indicated (dashed line). (PDF) [file ppat.1006825.s007.pdf]
