# Supplementary material for: Phenotypic deficits in the HIV-1 envelope are associated with the maturation of a V2-directed broadly neutralizing antibody lineage
Source: PLoS Pathog. 2018 Jan 25;14(1):e1006825. doi: 10.1371/journal.ppat.1006825 (PMC5806907; doi:10.1371/journal.ppat.1006825)
Supplement: S1 Table — Overview of CAPR256 Env variants used in the current study. (PDF) [file ppat.1006825.s008.pdf]

Longitudinal CAP256 Env panel

Envelope clone	Classification based on V2 epitope	Epitope aa sequence [1]	GenBank accession number
6-wk[PI]	Primary virus (PI)	**NTITEVRDKQKKE**	KF996578
15-wk[SU]	Superinfecting virus (SU)	**NATTELRDKKKKE**	KF996583
23-wk.16[PI]	PI-like	**NTITEVRDKQKKE**	KF996596
30-wk.8[PI]	PI-like	**NTITEVRDKQKRE**	KF996610
34-wk.18[PI]	PI-like	**NTITEVRDKQKKE**	KF996623
34-wk.31[SU]	SU-like	**NATTELRDKIKKE**	KF996641
34-wk.77[SU]	SU-like	**NTTTELRDKKKKQ**	KT698225
34-wk.81[SU]	SU-like	**NATTELRDKKRRE**	KT698227
42-wk.5[SU]	SU-like	**NATTELRDKQKKE**	MF572813
42-wk.16[PI]	PI-like	**NTITEVRDKQKKE**	MF572821
42-wk.18[SU]	SU-like	**NATTELRDKTRRE**	MF572823
42-wk.24[SU]	SU-like	**NATTELRDKRRKE**	MF572829
48-wk.8[SU]	SU-like	**NATTELRDKIRKE**	KF996656
48-wk.17[PI]	PI-like	**NTITEVRDKQKKE**	KF996662
176-wk.C2[PI]	PI-like	**NTITEVSDKQKNE**	KF996701
176-wk.F1	PI/SU-recombinant	**NATTEVRDKEKKE**	KF996712
176-wk.4	PI/SU-recombinant	**NATTEVRDKEKKE**	KF996713

[1]Sequence data derived from Doria-Rose et al, 2015.
