# Supplementary material for: Phenotypic deficits in the HIV-1 envelope are associated with the maturation of a V2-directed broadly neutralizing antibody lineage
Source: PLoS Pathog. 2018 Jan 25;14(1):e1006825. doi: 10.1371/journal.ppat.1006825 (PMC5806907; doi:10.1371/journal.ppat.1006825)
Supplement: S2 Table — Overview of CAP256-VRC26 bnAbs used in the current study. (PDF) [file ppat.1006825.s009.pdf]

CAP256-VRC26 bnAb characteristics and autologous free virus neutralization

Autologous free virus neutralization, IC50 [μg/ml] [1]

			PI-like viruses							SU-like viruses								PI/SU recombinant viruses		
bnAbs	Heterologous breadth [%][1]	AA mutations heavy chain from UCA [%][2]	6-wk[PI]	23-wk.16[PI]	30-wk.8[PI]	34-wk.18[PI]	42-wk.16[PI]	48-wk.17[PI]	176-wk.C2[PI]	15-wk[SU]	34-wk.31[SU]	34-wk.77[SU]	34-wk.81[SU]	42-wk.5[SU]	42-wk.18[SU]	42-wk.24[SU]	48-wk.8[SU]	176-wk.F1	176-wk.4	Autologous breadth [%][3]
**VRC26.12**	7	25.7		1.6360	0.6430	0.5243	9.0680			0.0310		0.0087	0.0077			0.0124				44
**VRC26.07**	13	25.3			1.6050					0.0027	0.2090	0.0027	0.0020	0.3101	1.4600	0.0026	0.5799			50
**VRC26.21**	13	32.2			6.1170					0.0009	0.0079	0.0016	0.0012	0.0042	0.0098	0.0004	0.0036			50
**VRC26.06**	17	22.9	0.0021	0.0031	0.0145	0.0013	0.0801	7.4980		0.0212	0.0148	0.0029	0.0140	4.4880	2.7980	0.0240	0.0243			78
**VRC26.01**	20	20.8								0.0599		0.0121	0.0389			2.3420				22
**VRC26.31**	20	29.5								0.0037	0.1386	0.0010	0.0065	0.2525	2.1160	0.0012	0.3417			44
**VRC26.05**	22	23.4								0.0066	0.1117	0.0008	0.0057	0.0952	1.9920	0.0019	0.4459			44
**VRC26.10**	24	27.8	7.5180							0.0066	0.3123	0.0093	0.0262	0.5619	2.8350	0.0054	0.7466			50
**VRC26.17**	28	22.9								0.0012	0.0106	0.0017	0.0042	0.0963	0.0433	0.0010	0.0193			44
**VRC26.08**	46	28.8			6.3880					0.0009	0.0045	0.0031	0.0003	0.0267	0.0341	0.0009	0.0620			50
**VRC26.09**	46	22.9			5.0370					0.0009	0.0024	0.0009	0.0011	0.0098	0.0466	0.0010	0.0794			50
**VRC26.25**	63	22.8	1.9470	0.4936	0.0006	0.1081	0.4405	0.2644		0.0012	0.0017	0.0004	0.0009	0.0043	0.0050	0.0004	0.0019			78

[1] IC_{50} obtained from autologous free virus neutralization assays on A3.01-CCR5 target cells.

[2] Data for heterologous breadth and amino acid mutations in the heavy chain from the unmutated common ancestor as published in Doria-Rose et al, 2015.

[3] The autologous breadth is based on the neutralization of the autologous viruses listed in this table.

IC50 [μg/ml] <0.01 >0.01, <1 >1 Not sensitive
