# Supplementary material for: Phenotypic deficits in the HIV-1 envelope are associated with the maturation of a V2-directed broadly neutralizing antibody lineage
Source: PLoS Pathog. 2018 Jan 25;14(1):e1006825. doi: 10.1371/journal.ppat.1006825 (PMC5806907; doi:10.1371/journal.ppat.1006825)
Supplement: S3 Table — Comparison (Mann-Whitney test) of sensitivity of VRC26 sensitive and early escape SU-like viruses to heterologous bnAbs directed to different epitopes. (PDF) [file ppat.1006825.s010.pdf]

Comparison heterologous bnAb neutralization of VRC26 sensitive and early escape SU-like viruses

**bnAb epitope**	**SU-like VRC26 sensitive viruses vs. VRC26 early escape viruses[1]**<br>**Comparison of bnAb activity[2]**<br>**(Mann-Whitney, p value)**	
	**Free virus**	**Cell-cell**
**CD4 binding site**	n.s. (0.3132)	**0.0225**
**V3 glycan**	n.s. (0.3929)	n.s. (0.7857)
**V2 apex**	n.s. (0.1292)	**0.0160**
**MPER**	n.s. (>0.9999)	**0.0357**

[1] Comparison between SU-like VRC26 sensitive (15-wk^{SU}, 34-wk.31^{SU}, 34-wk.77^{SU}, 34-wk.81^{SU}, 42-wk.24^{SU}) and SU-like VRC26 early escape (42-wk.5^{SU}, 42-wk.18^{SU}, 48-wk.8^{SU}) viruses

[2] Heterologous bnAb data depicted in Fig 2A and 2B were compared. SU-like VRC26 early escapes were significantly less sensitive to CD4bs, V2 apex and MPER bnABs
